# Long-Term Outcomes of Single and Dual Anastomosis Duodenal Switch

**DOI:** 10.1007/s11695-025-08114-x

**Published:** 2025-08-09

**Authors:** Ana Marta Pereira, Sofia S. Pereira, Mário Nora, Rui F. Almeida, Mariana P. Monteiro, Marta Guimarães

**Affiliations:** 1 Department of General Surgery, Unidade Local de Saúde de Entre o Douro e Vouga, Santa Maria da Feira, Portugal; 2 School of Medicine and Biomedical Sciences (ICBAS), University of Porto, Porto, Portugal; 3 Unit for Multidisciplinary Research in Biomedicine (UMIB), Porto, Portugal

**Keywords:** Biliopancreatic diversion with duodenal switch (BPD/DS), Single anastomosis duodenoileal bypass with sleeve gastrectomy, Long-term safety profiles, QoL

## Abstract

**Background:**

Single anastomosis duodenal-ileal bypass with sleeve gastrectomy (SADI-S) offers a streamlined alternative to biliopancreatic diversion with duodenal switch (BPD/DS), potentially with a lower risk of complications in patients with obesity grade III, although long-term comparative studies are lacking.

**Purpose:**

To compare long-term outcomes of patients undergoing SADI-S and BPD/DS.

**Methods:**

A cohort of 114 patients with a body mass index (BMI) equal to or greater than 45 kg/m^2^ who underwent BPD/DS or SADI-S in a single bariatric public center as a primary intervention between 2015 and 2019 was evaluated for a follow-up period of at least 60 months.

**Results:**

After ≥ 60 months of follow-up, patients submitted to BPD/DS and SADI-S achieved a total weight loss (TWL) > 20% (96% vs 91%, *p* = 0.67) and similar remission rates of associated medical problems.

Transient vitamin and micronutrient deficiencies during follow-up were observed in 44.8% of BPD/DS patients and 63.5% of SADI-S patients, anemia in 44.8% and 42.4%, and iron deficiency in 58.6% and 48.2%, respectively. Quality of life (QoL) scores were not significantly different between the groups (BPD/DS: 2.00 ± 0.22 vs. SADI-S: 2.15 ± 0.19, *p* = 0.08). After propensity score matching (*n* = 28 per group), differences in weight loss outcomes became more pronounced, favoring BPD/DS, while SADI-S was associated with significantly greater improvement in quality-of-life.

**Conclusions:**

The long-term outcomes of BPD/DS and SADI-S in terms of obesity-related comorbidities remission and complication rates do not seem to differ, despite BPD/DS inducing greater weight loss and SADI-S being associated with greater improvements in quality of life.

**Supplementary Information:**

The online version contains supplementary material available at 10.1007/s11695-025-08114-x.

## Introduction/Purpose

Biliopancreatic diversion with duodenal switch (BPD/DS) remains the most effective weight loss procedure [1]. Surgical refinements have reduced its complexity and early complication rates [2]. Nonetheless, it accounts for under 2% of metabolic bariatric surgeries globally [3], largely due to concerns about long-term nutritional risks and potential quality of life (QoL) impairment [4, 5].

Single anastomosis duodenoileal bypass with sleeve gastrectomy (SADI-S) has emerged as a streamlined alternative, preserving the core principles of BPD/DS [6]. It provides comparable restriction through sleeve gastrectomy (SG), a similar total absorptive limb, and a post-pyloric anastomosis. Operative time is reduced by eliminating one anastomosis and the need to close a mesenteric defect [7–9]. It is feasible in two stages and serves as an alternative for conversional surgery following suboptimal outcomes after SG [10]. A key advantage is the lower expected risk of long-term complications—particularly nutritional deficiencies—due to its longer common channel (CC) [7, 8]. Although robust clinical evidence is lacking, higher postprandial amino acid levels in SADI-S compared to BPD/DS support a potential nutritional benefit [11]. Whether this advantage extends to other nutrients, especially regarding fat-soluble vitamins, which rely on prolonged contact between food and biliopancreatic secretions for optimal absorption, remains unclear.

SADI-S elicits a distinctive postprandial endocrine response, notably a more pronounced glucagon-like peptide-1 (GLP-1) release compared to BPD/DS, similar to that observed in Roux-en-Y gastric bypass (RYGB) with a longer biliopancreatic limb (BPL). Greater glycemic variability has also been reported following SADI-S [12].

Several studies have compared SADI-S and BPD/DS short- and mid-term outcomes, showing broadly comparable results [7, 9, 13–15]. However, in the absence of direct long-term comparisons, the clinical significance of these differences remains uncertain.

This study aimed to assess and compare the long-term outcomes of patients undergoing SADI-S or BPD/DS. Primary endpoints included weight loss and resolution of obesity associated medical problems, while secondary outcomes comprised complication rates, nutritional deficiencies and QoL.

## Material and Methods

This retrospective comparative analysis of prospectively followed cohorts was conducted at a single public bariatric surgery center and approved by the Institutional Ethical Review Board. The STROBE checklist guided the study design and reporting.

A total of 114 patients underwent SADI-S or BPD/DS between February 2015 and December 2019. Supplementary Fig. 1 outlines the patient selection process. Short- and mid-term outcomes, surgical techniques, and follow-up protocols for this cohort were previously reported [9]. Briefly, patient visits were scheduled per study protocol up to 48 months after surgery, while the 60-month visit represents an extension approved through a study protocol addendum. At this time point, all patients were invited for an additional follow-up appointment.

A body mass index (BMI) of 45 kg/m^2^ or greater was established as eligibility criteria to undergo hypoabsorptive procedures, such as SADI-S or BPD/DS. The allocation to each surgical technique was based on ethically sound and clinically justifiable criteria, namely: (1) Risk of Nutrient Deficiencies: Patients with borderline nutritional status, previous history of micronutrient deficiencies, financial constraints or inability to comply with long-term follow-up were allocated to SADI-S, which were identified as risk factors for nutritional deficiencies; (2) Patient Preference and Shared Decision-Making: the final choice of procedure incorporated patient preferences, following detailed information on the technical details, as well as potential risks and advantages of each procedure; (3) Anatomical or Technical Considerations: identified at preoperative imaging or intraoperative findings (e.g., previous surgeries, adhesions, or intestinal length variation) that favor one technique over the other.

Exclusion criteria included severe gastroesophageal reflux disease (GERD) unresponsive to proton pump inhibitors (PPI), presumed failure to comply with micronutrient supplements, prior metabolic bariatric surgeries or endoscopic weight loss procedures. Preoperative GERD was diagnosed by upper endoscopy-confirmed esophagitis alongside a suggestive clinical history. Esophageal impedance was performed when alkaline reflux was suspected. In such cases, BPD/DS was preferred, assuming pyloric dysfunction.

Weight trajectories were assessed using BMI, percentage (%) total weight loss (%TWL), and % excess BMI loss (%EBMIL). Follow-up obesity associated medical problems included type 2 diabetes (T2D), hypertension (HT), dyslipidemia (DL), and metabolic syndrome (MS), defined by harmonized criteria [16], with outcomes reported per Brethauer et al. [17].

In patients undergoing SADI-S with total bowel length measurement, the correlation between BPL length and weight loss was assessed. Secondary endpoints included short-term (≤ 90 days) and long-term (> 90 days) complications, classified using Clavien-Dindo (CD) [18], nutrient deficiencies (total proteins, ferritin, iron, vitamin D, and calcium at any follow-up point), and QoL assessed at the end of follow-up using the Bariatric Analysis and Reporting Outcome System (BAROS) questionnaire [19]. Late morbidity was defined as complications requiring acute intervention, such as surgery or hospitalization. Esophageal impedance was performed in patients with PPI-refractory GERD symptoms to detect non-acid reflux, while upper endoscopy ruled out GERD-related lesions. Daily bowel movement frequency was also recorded.

## Statistical Analysis

Continuous variables are expressed as means ± standard errors, and categorical variables as counts and percentages, unless otherwise specified. Categorical comparisons were made using Fisher’s exact test. A linear regression analysis was performed to evaluate the impact of sex, age, BMI, and presence of T2D before surgery on the primary outcome, % TWL after surgery. Additionally, linear regression was also used to assess the predictive value of BPL length for %TWL following SADI-S surgery.

Postoperative BMI, weight, %TWL, and %EBMIL were compared between surgical groups using ANCOVA, adjusting for the covariates sex, age, preoperative BMI, and presence of T2D.

In addition, a subgroup analysis was conducted using propensity score matching based on sex, preoperative age, body mass index (BMI), and Type 2 Diabetes (T2D) status, resulting in 28 patients per group. Matching was performed using a 1:1 nearest-neighbor approach and a caliper of 0.1 on the propensity score.

To address missing data in our primary outcome (%TWL) at multiple follow-up time points, we performed multiple imputation using SPSS’s Multiple Imputation procedure. %TWL were imputed simultaneously, including relevant predictor variables such as age, sex, surgery type, baseline BMI, and T2D status. Five imputed datasets were generated, and subsequent analyses were conducted on the pooled results to appropriately account for uncertainty due to missing data. Results after imputation were consistent with the complete-case analysis, preserving the direction and significance of group differences.

A Kaplan–Meier survival analysis was used to assess time to first early postoperative complication (≤ 90 days). Group comparisons were made using the Log-rank (Mantel-Cox) test.

Normality was assessed with the Kolmogorov–Smirnov test. Baseline continuous variables and laboratory data were compared between groups using unpaired Student’s t-test or Mann–Whitney U test, based on the normality test results. Statistical significance was set at p < 0.05. Analyses were performed with GraphPad Prism v10.4.1 and SPSS v29. Post-hoc power analysis for %TWL was calculated using the PS Power and Sample Size Calculations software, for Windows.

## Results

Between February 2015 and December 2019, a total of 129 patients underwent SADI-S or BPD/DS. Fifteen patients who underwent revisional procedures were excluded. Of the remaining 114 patients, 29 underwent BPD/DS and 85 underwent SADI-S, with a pre-operative BMI of 53.41 ± 0.93 and 50.62 ± 0.51 kg/m^2^, respectively. At 60 months, 25 patients (86.2%) in the BPD-DS group and 67 patients (78.8%) in the SADI-S group completed the 60-month follow-up assessment. The average follow-up time was 83.56 ± 1.81 months; the preoperative patient characteristics are shown in Table 1.

**Table 1:** Demographic, anthropometric and clinical data before surgery

	Surgery		
	BPD-DS	SADI-S	*p*
*n* (%)	29 (25%)	85 (75%)	-
Demographic, anthropometric data			
Age, years	40.55 ± 2.12	42.60 ± 1.24	0.432
Sex n. M/F (%)	8/25 (24.2/75.8)	27/97 (21.8/78.2)	
Weight, kg	142.10 ± 4.36	132.50 ± 1.93	**0.043**
BMI, kg/m^2^	53.41 ± 0.93	50.62 ± 0.51	**0.004**
% EBMI	28.41 ± 0.93	25.62 ± 0.51	**0.004**
Comorbidities, %			
T2D	8 (27.6%)	16 (18.8%)	0.429
Dyslipidemia	19 (65.5%)	51 (60.0%)	0.663
HT	12 (41.4%)	38 (44.7%)	0.830
MS	15 (51.7%)	43 (50.6%)	1.000
Sleep Apnea	4 (13.8%)	18 (21.2%)	0.586
Osteoarthritis	8 (27.6%)	23 (27.1%)	1.000
GERD	7 (24.1%)	16 (18.8%)	0.595

BMI, body mass index; %EBMIL, percentage of excess of BMI loss; T2D, type 2 diabetes; HT, hypertension; MS, metabolic syndrome; GERD, gastroesophageal reflux disease; BPD/DS, biliopancreatic diversion with duodenal switch; SADI-S, single anastomosis duodeno-ileal with sleeve gastrectomy. Fisher’s exact test was used to compare nominal variables and unpaired Student’s t-test or Mann–Whitney U test was used to compare continuous variables between the surgical groups. Significant differences at bold

### Weight Loss

**Fig. 1:**
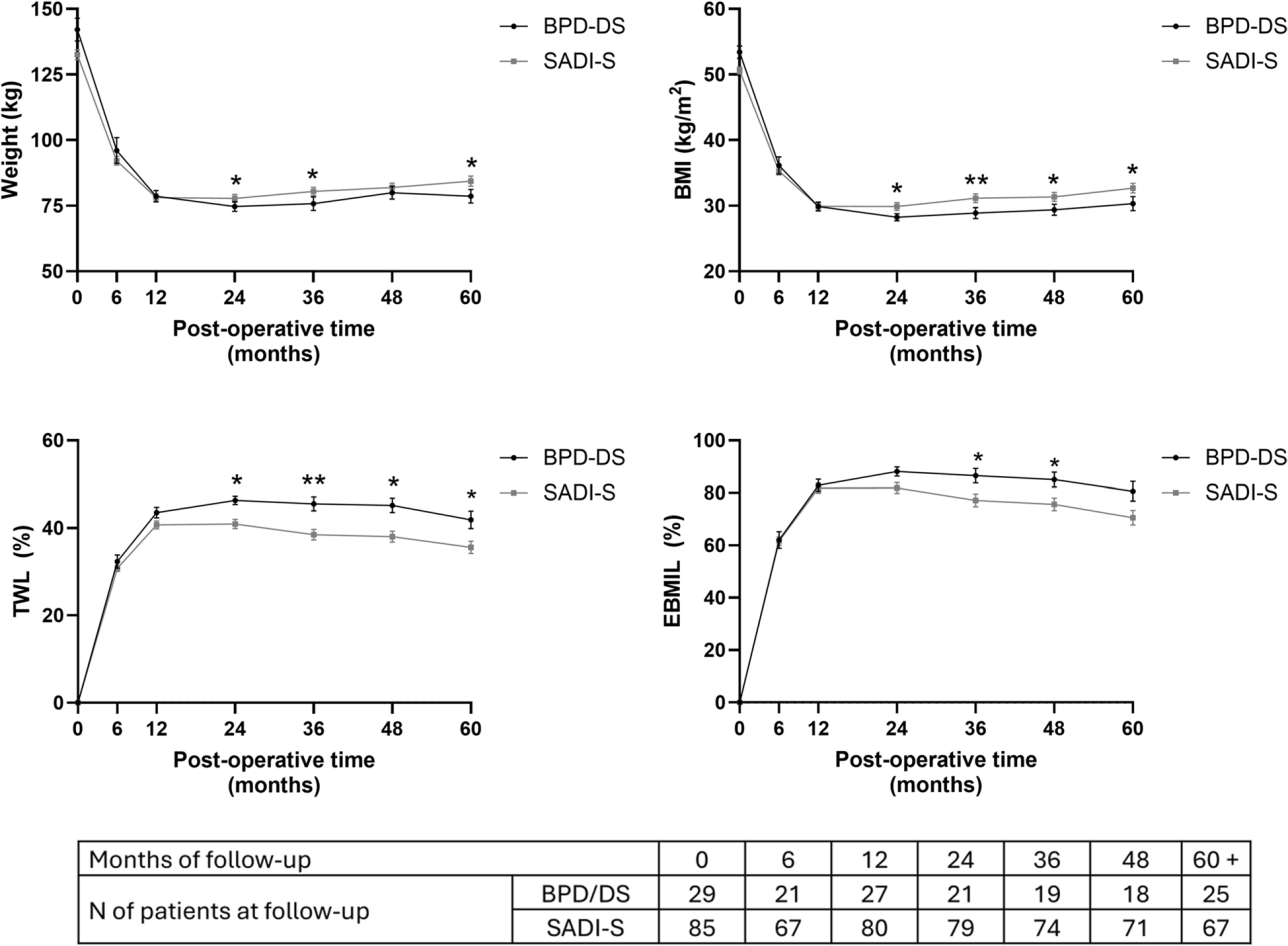
Graphic representation of weight, body mass index (BMI), percentage of total weight loss (%TWL) and percentage of excess of BMI loss (%EBMIL) of patients submitted to biliopancreatic diversion with duodenal switch (BPD/DS) and single anastomosis duodenal-ileal with sleeve gastrectomy (SADI-S) during the follow-up of ≥ 60 months. No statistically significant differences were observed between groups (*p* > 0.05) for weight and BMI. However, differences in %TWL emerged at 24 months and remained significant thereafter, while differences in %EBMIL became significant at 48 months and continued to be significant afterward (*ANCOVA adjusted for sex, age, preoperative BMI, and presence of T2D. Significance: p* < 0.05*; *p* < 0.01**)

Age, sex, and BMI at the time of surgery significantly influenced %TWL at specific postoperative time points, regardless of the surgical procedure (Supplementary Table 1). Both procedures led to substantial weight reduction during the first postoperative year, followed by sustained stability. BPD/DS showed a slight but statistically significant advantage in %TWL after the second year, although this difference decreased over time. At ≥ 60 months, 96% of BPD/DS patients and 91% of SADI-S patients maintained a TWL over 20%, with no significant difference between groups. Weight loss trajectories are depicted in Fig. 1 and detailed in Supplementary Table 2. After propensity score matching, the differences between BPD/DS and SADI-S became more pronounced, demonstrating that patients submitted to BPD/DS achieved a greater weight loss (Supplementary file 1).

**Fig. 2:**
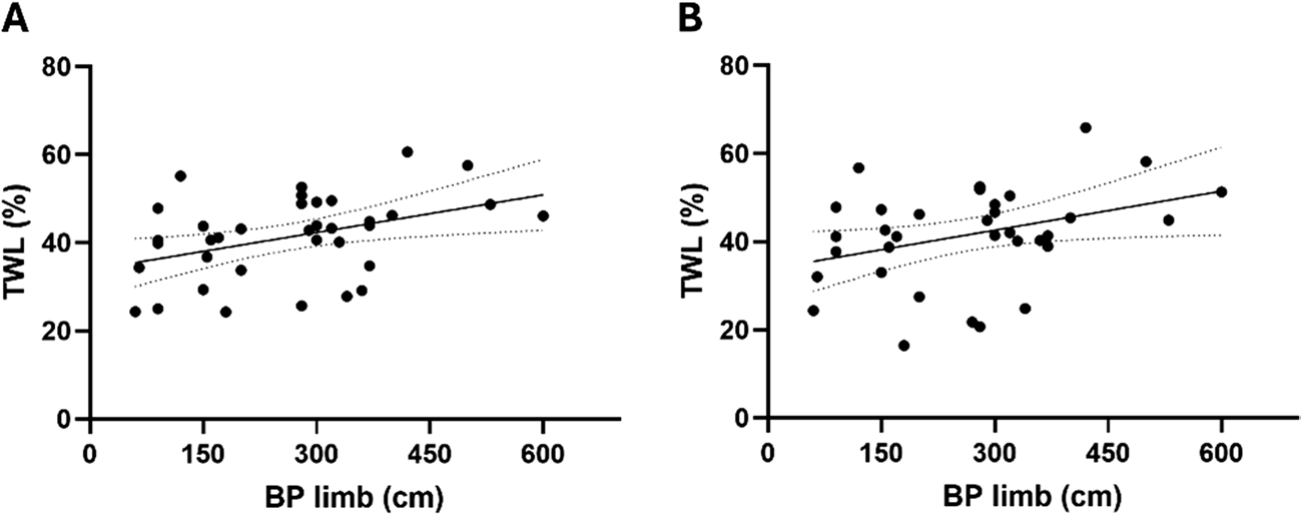
Correlation between biliopancreatic limb (BPL) length and total weight loss (TWL) at 12 (**A**) and 24 (**B**) months after surgery. BPL size significantly predicted the %TWL 12 and 24 months after surgery (Linear regression, *p < *0.05)*

Total limb length was measured in 37 patients submitted to SADI-S. BPL length significantly predicted the short-term %TWL 12 months (HR 0.029 [95% CI 0.006; 0.051], *p* = 0.014) and 2 years after surgery (HR 0.030 [95% CI 0.002; 0.051], *p* = 0.057), in a magnitude of 0.3% greater %TWL for each 10 cm longer BPL (Fig. 2).

### Obesity Associated Medical Problems

Remission rates of obesity associated medical problems did not differ significantly between groups (Supplementary Table 3). Both surgeries improved glucose and lipid profiles, with no significant differences at ≥ 60 months, except for a greater reduction in total cholesterol with BPD/DS (Supplementary Table 4). Additionally, no significant differences in obesity associated medical problems remission rates were observed after propensity score matching (Supplementary file 1).

### Complications and Nutritional Deficiencies

No significant differences in early (≤ 90 days) or late (> 90 days) morbidity were observed between groups (Table 2 and Supplementary Fig. 2). No mortality occurred.

**Table 2:** Postoperative short and long-term complications

	BPD-DS	SADI-S	*p*
Mortality	0 (0.0%)	0 (0.0%)	-
Early morbidity, ≤ 90 d	4 (13.8%)	11 (12.9%)	> 0.999
Late morbidity, > 90 d	3 (10.3%)	4 (4.7%)	0.370
Reintervention	5 (17.2%)	6 (7.1%)	0.144
Clavien-Dindo Classification (≤ 90 d)			
Grade I	0 (0.0%)	0 (0.0%)	0.754
Grade II	1 (3.5%)	3 (3.5%)	
Grade IIIa	0 (0.0%)	2 (2.4%)	
Grade IIIb	3 (10.3%)	4 (4.7%)	
Grave IVa	0 (0.0%)	2 (2.4%)	
Grade IVb	0 (0.0%)	0 (0.0%)	
Complications ≤ 90d			
Leaks	0 (0.0%)	4 (4.7%)	0.293
Peritonitis	0 (0.0%)	1 (1.2%)	> 0.999
Intraabdominal abscess	1 (3.5%)	6 (7.1%)	0.676
Hemoperitoneum	0 (0.0%)	1 (1.2%)	> 0.999
Digestive Bleeding	1 (3.5%)	0 (0.0%)	0.254
Port herniation	1 (3.5%)	0 (0.0%)	0.254
Wound infection	0 (0.0%)	1 (1.2%)	> 0.999
Pulmonary infection	0 (0.0%)	1 (1.2%)	> 0.999
Pulmonary embolism	0 (0.0%)	2 (2.4%)	> 0.999
Admission for severe nutritional deficiencies	1(3.5%)	2 (2.4%)	> 0.999
Complications > 90d			
Internal hernia	3 (10.3%)	1 (1.2%)	0.050
Vitamin deficiencies	13 (44.8%)	54 (63.5%)	0.086
Neurologic disorder for vitamin deficits	0 (0.0%)	2 (2.4%)	> 0.999
Anemia	13 (44.8%)	36 (42.4%)	0.430
Iron-deficiency anemia	17 (58.6%)	41 (48.2%)	0.393
GERD	6 (20.7%)	30 (35.3%)	0.571
Biliary reflux	0 (0.0%)	4 (4.7%)	0.293
Dumping Symptoms	1 (3.5%)	7 (8.2%)	0.677

Fisher’s exact test was used to compare complications between the surgical groups

BPD/DS, biliopancreatic diversion with duodenal switch; SADI-S, single anastomosis duodeno-ileal with sleeve gastrectomy; GERD, gastroesophageal reflux disease

Two SADI-S patients were hospitalized for neurological disorders due to vitamin B1 and A deficiencies, both linked to non-compliance with micronutrient supplementation recommendations. One of the patients recovered completely without sequelae, the other had permanent ophthalmoplegia. In addition, one BPD/DS patient was admitted with anasarca from severe protein deficiency after viral gastroenteritis, successfully managed with enteral and parenteral nutrition. All cases were treated medically, without the need for surgical revision or reversal. Transient vitamin/micronutrient deficiencies occurred in 44.8% (BPD/DS) and 63.5% (SADI-S); anemia in 44.8% and 42.4%; and iron deficiency in 58.6% and 48.2%, respectively. Transient vitamin deficiencies were more frequent after SADI-S. Nutritional status is shown in Supplementary Tables 4 and 5.

GERD symptoms were reported in 20.7% of BPD/DS and 35.3% of SADI-S patients during follow-up. At ≥ 60 months, prevalence dropped to 15.8% and 23.8%, respectively, with no significant difference. Among 23 patients symptomatic preoperatively, only 2 (BPD/DS) and 6 (SADI-S) remained symptomatic. De novo GERD occurred in 28 patients (4 BPD/DS, 24 SADI-S). Biliary reflux was confirmed in 4 SADI-S patients, with 2 requiring conversion to BPD/DS due to refractory symptoms.

Dumping symptoms during follow-up occurred in 1 patient submitted to BPD/DS (3.5%) and 7 submitted to SADI-S (8.2%), all managed with dietary counseling. Postoperative bowel movement frequency remained stable (1–2/day), with no significant group differences or QoL impact. Asymptomatic biliary lithiasis was identified preoperatively in 3 BPD/DS and 17 SADI-S patients. Although no cholecystectomies were performed concurrently, subsequent surgery was required in 1 BPD/DS and 4 SADI-S patients, respectively, due to symptoms or complications.

### Quality of Life

The BAROS QoL questionnaire was completed by 66 patients with ≥ 60 months of follow-up (12 BPD/DS; 54 SADI-S), as detailed in Table 3. There was no significant difference in mean QoL scores between groups (BPD/DS: 2.00 ± 0.22 vs. SADI-S: 2.15 ± 0.19, *p* = 0.08). Most patients in both groups reported their QoL as “Improved” or “Greatly Improved,” and the distribution of these categorical responses was not statistically different in the unmatched analysis. However, after propensity score matching, significant differences in QoL categories became evident favoring greater improvements after SADI-S (Supplementary file 1).

**Table 3:** Quality of life questionnaire scores 60 + months after surgery

Self Esteem		*Much Worse*	*Worse*	*The Same*	*Better*	*Much Better*
	BPD-DS	0.0%	0.0%	0.0%	50.0%	50.0%
	SADI-S	0.0%	5.6%	5.6%	16.7%	72.2%
Physical		*Much Less*	*Less*	*The Same*	*More*	*Much More*
	**BPD-DS**	0.0%	0.0%	0.0%	50.0%	50.0%
	**SADI-S**	0.0%	5.6%	5.6%	20.4%	68.5%
Social		*Much Less*	*Less*	*The Same*	*More*	*Much More*
	**BPD-DS**	0.0%	0.0%	0.0%	50.0%	50.0%
	**SADI-S**	0.0%	7.4%	11.1%	11.1%	70.4%
Labor		*Much Less*	*Less*	*The Same*	*More*	*Much More*
	**BPD-DS**	0.0%	0.0%	8.3%	41.7%	50.0%
	**SADI-S**	0.0%	7.4%	14.8%	11.1%	66.7%
Sexual		*Much Less*	*Less*	*The Same*	*More*	*Much More*
	**BPD-DS**	0.0%	0.0%	72.7%	18.2%	9.1%
	**SADI-S**	1.9%	3.8%	32.7%	3.8%	57.7%
Scoring		*Greatly Diminished*	*Diminished*	*Minimal to no change*	*Improved*	*Greatly Improved*
	**BPD-DS**	0.0%	0.0%	0.0%	45.5%	54.5%
	**SADI-S**	0.0%	5.8%	7.7%	15.4%	71.2%
	**p**	0.213				

Fisher’s exact test was used to compare the quality-of-life score between the surgical groups. BPD/DS, biliopancreatic diversion with duodenal switch; SADI-S, single anastomosis duodeno-ileal with sleeve gastrectomy

## Discussion/Conclusion

The efficacy of BPD/DS and SADI-S was previously compared by several studies, given its anatomical similarities [8, 14, 15]. SADI-S is often favored for its technical simplicity and anticipated reduction in the risk of long-term complications, potentially making it the preferred approach for grade III obesity or higher. However, this hypothesis still needed to be confirmed by long-term studies, which was the primary aim of this study. After ≥ 60 months of follow-up, both procedures showed comparable resolution of obesity-associated medical problems and complication rates, despite BPD/DS depicting a numerical advantage in total weight loss over SADI-S, at the expense of a disadvantage in QoL outcomes.

### Efficacy

Both procedures effectively promote weight loss, with over 90% of patients meeting the TWL > 20% success criterion at all time points and %EBMIL exceeding 70% one-year post-surgery. Although BPD/DS depicted a numerical advantage for TWL, the remission rate of associated medical problems was similar. Even though long-term outcomes after BPD/DS are well documented [5], there is a single report of SADI-S outcomes beyond ten years[20], while comparative analyses are limited to two prospective studies spanning five years or more [14, 15] and one retrospective study exceeding five years [8], denoting a need for additional evidence to support the clinical decision making when choosing between procedures. Our findings are consistent with the previous studies’ data, confirming the superior TWL outcomes for BPD/DS.

Of particular note, a significant correlation between BPL length and TWL was observed in the SADI-S group during the first two postoperative years. Although the small number of patients with BPL data limits assessment of its long-term impact. Early weight loss has recognized prognostic value [21] and T2D remission is linked to weight loss within the first two years [22]. In DS-based procedures like BPD/DS and SADI-S, emphasis is typically placed on CC length due to its relevance for nutritional risks [23], while BPL is often overlooked. Despite comparable total absorptive limb lengths, interindividual variability leads to differing BPL lengths, which may influence weight outcomes, as seen in RYGB [24, 25]. Although neither procedure was associated with suboptimal weight loss (TWL < 20%), these findings underscore the potential value of individualized tailoring and merit further investigation.

### Safety

The technical complexity of duodenal switch procedures, whether with one or two anastomoses, no longer impacts perioperative safety [2]. The rate of severe early complications (CD > II) in our study was low and aligns with previous reports [2, 7].

SADI-S avoids complications associated with the ileoileal anastomosis in BPD/DS, such as leaks, kinking, internal hernias, and intussusception [2, 26]. Although internal hernias were more frequent in BPD/DS, the difference was not significant, with only three cases reported. One Peterson’s hernia occurred after SADI-S [20].

Concerns about macro- and micronutrient deficiencies have limited the use of hypoabsorptive techniques [27]. While both procedures showed transient vitamin deficiencies, they were more common after SADI-S, whereas BPD/DS had higher rates of iron deficiency and anemia. Despite average vitamin levels generally favoring SADI-S, differences were minimal and deficiencies were reversible with oral supplementation.

Two patients submitted to SADI-S were hospitalized early after surgery due to neurologic symptoms related to severe deficiencies, highlighting the critical need for supplementation compliance during the rapid weight loss phase. Furthermore, long-term adherence to supplementation remains a concern: 25% of patients were noncompliant at ≥ 60 months, despite preoperative counseling. Compliance was not formally linked to deficiencies, but many patients only resumed supplementation after its diagnosis and often used inadequate, low-cost alternatives.

Protein deficiency requiring urgent nutritional intervention is a major concern following BPD. Scopinaro tailored stomach volume and the alimentary limb to patient characteristics [28], while the DS preserved the pylorus and vagal innervation to reduce dumping and diarrhea [29]. Despite these refinements, long-term protein and albumin deficiencies remained frequent, often necessitating revision or reversal surgery. Diarrhea and steatorrhea with incontinence—reported in over 50% of cases in some series—may further impair protein absorption, though without clear distinction between BPD, BPD/DS, or shorter CC lengths [26, 27]. Recent studies using limb configurations similar to those adopted at our center have reported a reduced need for reversal due to hypoalbuminemia or refractory diarrhea [7, 13], a trend also observed in SADI-S with a 300 cm CC [20].

In our herein study, patients had on average 1–2 bowel movements per day, and diarrhea was rare regardless the procedure. Dumping syndrome, which may result in osmotic diarrhea during its initial phase, was also infrequent.

One patient was hospitalized with severe protein deficiency triggered by viral gastroenteritis. Although rare, late-onset protein deficiency requiring parenteral nutrition despite prior normal levels has been reported after BPD/DS, particularly under physiological stress. While some protein digestion occurs via salivary amylase and in the alimentary limb, most amino acids are absorbed in the CC, where pancreatic enzymes act. Despite similar protein levels between groups, BPD/DS may carry a higher risk due to a shorter CC [11], especially when the intestinal mucosa is compromised. The colon may partly compensate for reduced small-intestinal absorption in malabsorptive states like BPD [28], but mucosal injury—as in gastroenteritis—can impair this mechanism.

GERD is a common concern in all procedures involving SG, with no expected difference between BPD/DS and SADI-S unless bile reflux is present—more likely after SADI-S. In our cohort, bile reflux was confirmed in four patients, though true incidence is uncertain as impedance testing was symptom-driven. GERD symptoms occurred more often following SADI-S, but without statistical significance, and most cases responded to PPIs. These findings align with previous reports [13].

### Quality of Life

Overall, the largest proportion of patients reported a significant improvement QoL, for all parameters except the sexual one, which depicted no significant variation after surgery. Notably, after propensity score matching, a significant improvement in the QoL sexual parameter after SADI-S when compared to BPD/DS. Notwithstanding, none of the patients submitted to BPD/DS attributed the lowest scores to any of the QoL metrics.

### Limitations

This study has several limitations to be acknowledged. As a retrospective analysis of a prospective cohort, it is subject to inherent biases. One of the main limitations of this study is the sample size imbalance between the surgical groups. This numerical imbalance is due to the fact that BPD/DS is technically more complex, time-consuming, and requires advanced laparoscopic expertise. Our center, by implementing both techniques at the same time and ensuring they were performed by the same team using a standardized technique in patients that were prospectively followed after surgery, reduced the bias derived from different learning curves.

The imbalance in patient numbers in the study groups resulted for the tendency to perform the SADI-S technique more often than BPD/DS, since it allowed the team to operate on more patients in a shorter period, in compliance with waiting list time limits. Additionally, patient financial constrains, affecting their ability to afford adequate protein ingestion, favored the choice of SADI-S due to its lower associated risk of nutrient deficiencies. This imbalance may have impacted the statistical power of some analyses, particularly for categorical variables analyzed with Fisher’s exact test. Although the Kolmogorov–Smirnov test was used to assess normality of continuous variables, the small sample size in the BPD/DS group may limit the robustness of this assessment. In addition, despite the adjusted analyses performed, these cannot fully account for non-random allocation. To strengthen our statistical analysis, we have also performed a 1:1 propensity score matching and found that the matched results were largely consistent with the full cohort analysis, except for a more pronounced weight loss observed in the BPD-DS group. These findings reinforce the robustness of the observed differences.

The 4-year recruitment period was necessary because of the low percentage of the population in our geographic region with a BMI over 45. This extended timeframe introduces the potential for temporal confounding due to external factors, such as changes in surgical experience over time. This limitation was mitigated by the fact that all surgeries were performed by three pairs of experienced surgeons from a single team, using standardized anesthetic and surgical protocols, which minimized inter-operator variability.

This retrospective comparative analysis of prospectively followed patients was not initially designed to assess the impact of biliopancreatic limb length. Nonetheless, in a subset of patients, the biliopancreatic limb length was documented, which allowed the analysis of this parameter in 37 out of the 85 patients submitted to SADI-S. While most patients were followed for 2–3 years, loss to follow-up increased thereafter, particularly among those living farther from the center, potentially introducing bias and affecting adherence to supplementation, despite guidance from general practitioners. To assess potential bias from differential follow-up, we compared baseline and postoperative characteristics between patients who attended the 60-month visit and those who did not, within each surgical group. To address the potential for bias due to differential loss to follow-up, we compared baseline and postoperative characteristics between patients who completed the 60-month follow-up and those who did not, stratified by surgical group. This analysis did not reveal any consistent or systematic pattern of bias that could favor one surgical group over the other.

We also addressed missing data using multiple imputation. The imputed results for %TWL demonstrated a low Fraction of Missing Information, a low Relative Increase in Variance, and Relative Efficiency values close to 1. These indicators suggest that the effect of missing data on our estimates was minimal, and the imputation procedure yielded reliable results that support the robustness of our findings.

Finally, a post-hoc power analysis for %TWL at medium- and long-term follow-up indicated power values ranging from 68 to 77%. These values, although acceptable, reflect a moderate risk of Type II error. These considerations should be taken into account when interpreting the findings.

Nonetheless, the study has important strengths. The outcomes of these procedures beyond five years had only been compared in two studies, and none was a long-term randomized trial. Although long-term outcomes of BPD and BPD/DS are well documented independently, the impact of recent technical refinements remains poorly understood. Despite comparable results across most outcomes, QoL—an essential treatment goal—remains underexplored. Furthermore, analysis of BPL length in SADI-S offers valuable insight into its potential influence on weight loss, warranting further investigation.

As conclusions, the long-term outcomes of BPD/DS and SADI-S appear to converge in terms of resolution of obesity associated medical conditions, complication rates, and nutritional status. BPD/DS was associated with greater weight loss over time, while SADI-S showed a greater improvement in QoL. Additionally, in the SADI-S group, BPL length influenced total weight loss in the first two years after surgery, suggesting a potential for optimizing the surgical outcomes. However, these findings must be interpreted with caution given the potential selection bias, and limited power for some subgroup analyses. Further prospective, randomized studies with larger cohorts and longer-term follow-up are necessary to validate these results.

## Supplementary Information

Below is the link to the electronic supplementary material.

ESM 1 (PNG 61.4 KB)

ESM 1 TIF (453 KB)

ESM 2 (PNG 38.9 KB)

ESM 2 TIF (288 KB)

ESM 3 DOCX (58.8 KB)

ESM 4 DOCX (16.8 KB)

ESM 5 DOCX (20.2 KB)

ESM 6 DOCX (20.9 KB)

ESM 7 DOCX (34.3 KB)

ESM 8 DOCX (20.4 KB)

## Data Availability

No datasets were generated or analysed during the current study.
