# Supplementary figures and images for: Long-Term Outcomes of Single and Dual Anastomosis Duodenal Switch
Source: Obes Surg. 2025 Aug 9;35(9):3791–800. doi: 10.1007/s11695-025-08114-x (PMC12457490; doi:10.1007/s11695-025-08114-x)

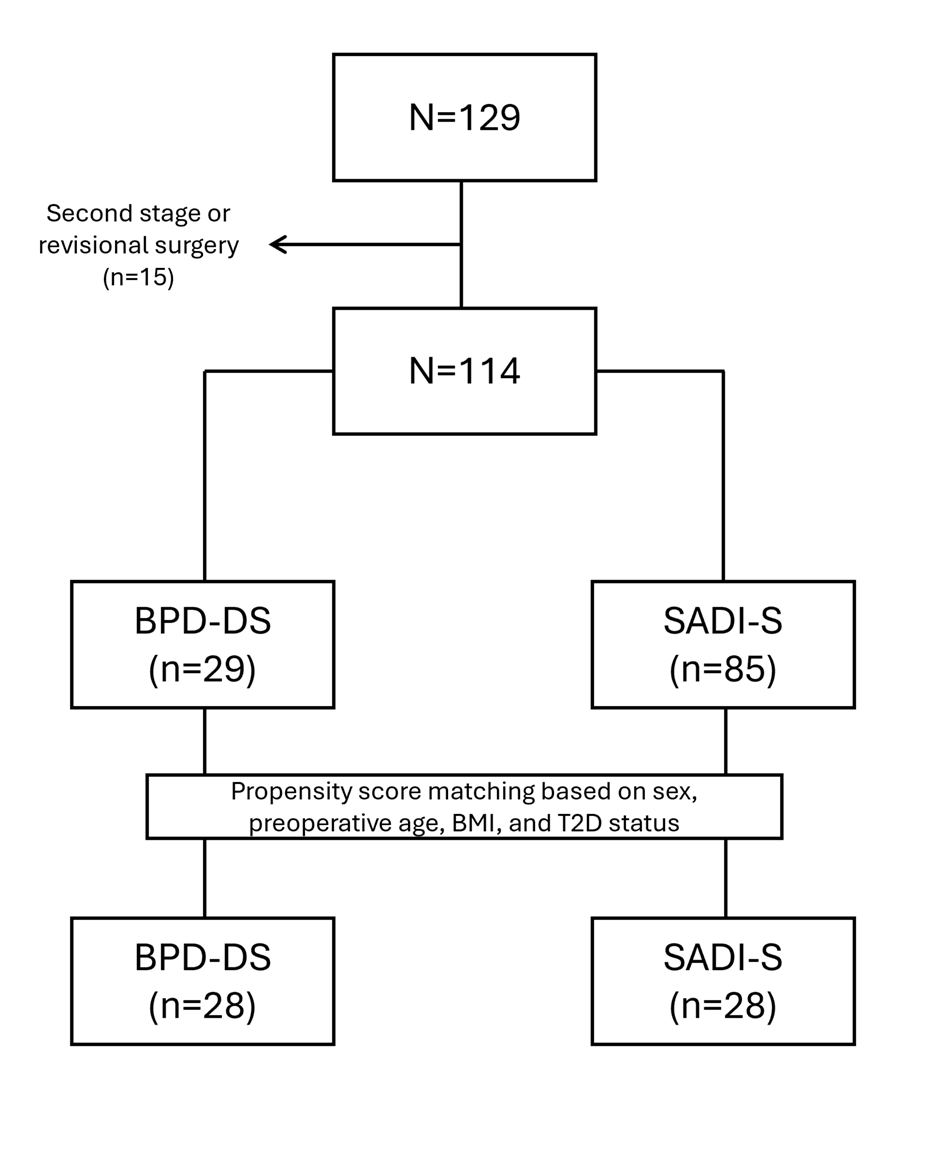

Supplement: Supplementary file 1 — (PNG 61.4 KB) [file 11695_2025_8114_Fig3_ESM.png]

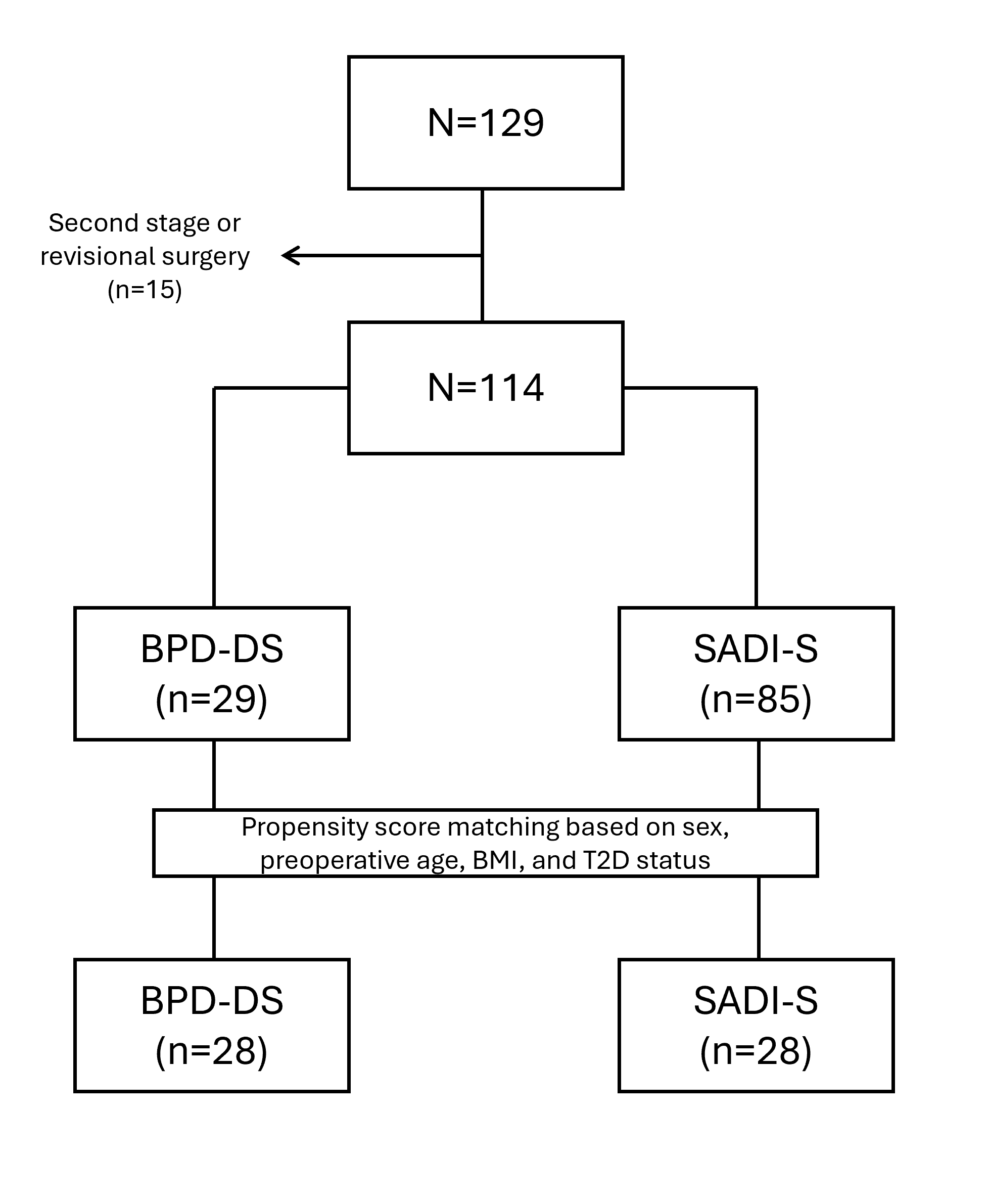

Supplement: Supplementary file 2 — TIF (453 KB) [file 11695_2025_8114_MOESM1_ESM.tif]

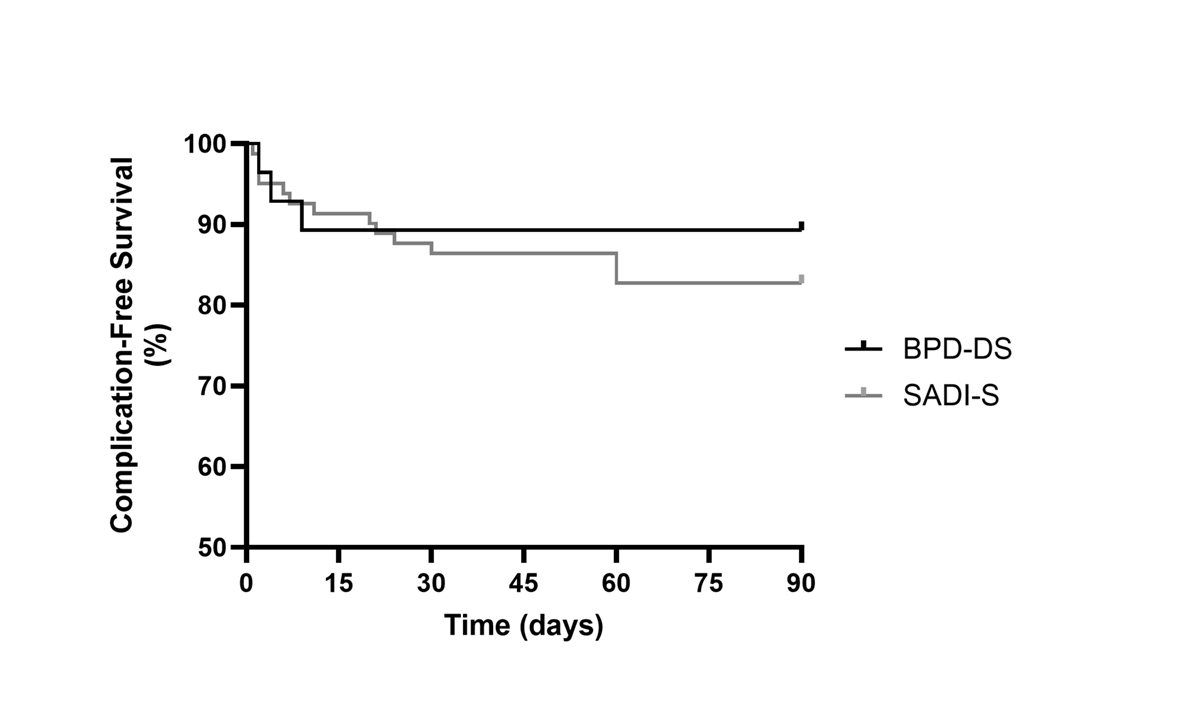

Supplement: Supplementary file 3 — (PNG 38.9 KB) [file 11695_2025_8114_Fig4_ESM.png]

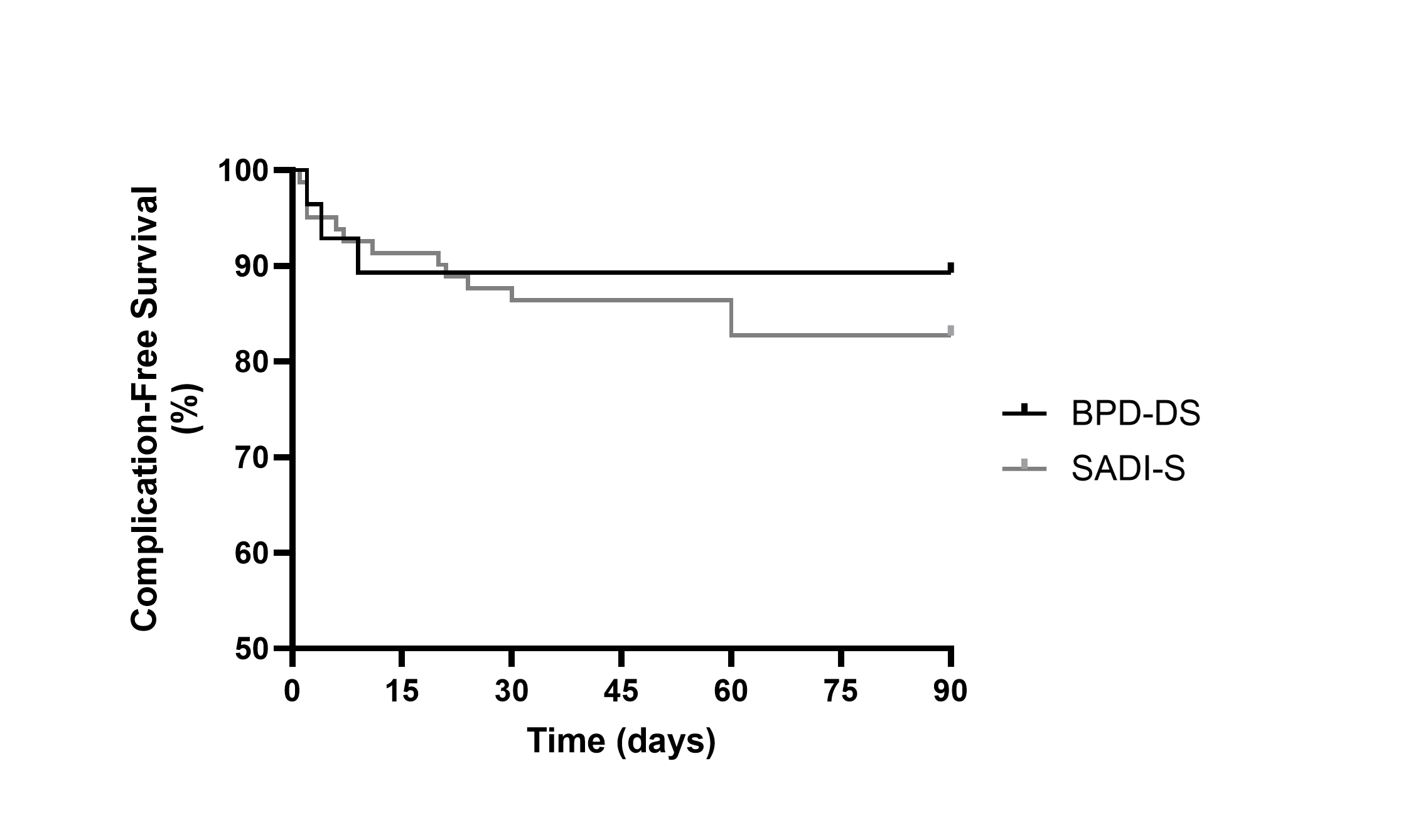

Supplement: Supplementary file 4 — TIF (288 KB) [file 11695_2025_8114_MOESM2_ESM.tif]
