# Supplementary material for: Long-Term Outcomes of Single and Dual Anastomosis Duodenal Switch
Source: Obes Surg. 2025 Aug 9;35(9):3791–800. doi: 10.1007/s11695-025-08114-x (PMC12457490; doi:10.1007/s11695-025-08114-x)
Supplement: Supplementary file 5 — DOCX (58.8 KB) [file 11695_2025_8114_MOESM3_ESM.docx]

**Supplementary File 1 – Propensity score matching analysis based on sex, preoperative age, body mass index, and Type 2 Diabetes status**

Table S1.1 Demographic, anthropometric and clinical data before surgery, after propensity score analysis.

|  |  | |  |
| --- | --- | --- | --- |
|  | **BPD-DS** | **SADI-S** | **p** |
| **n (%)** | 28 (50%) | 28 (50%) | - |
| **Demographic, anthropometric data** | | | |
| Age, years | 41.11 ± 2.12 | 40.46 ± 2.29 | 0.838 |
| Sex n. M/F (%) | 7/21 (25.0/75.0) | 7/21 (25.0/75.0) | 1.000 |
| BMI, kg/m^2^ | 52.84 ± 0.76 | 52.76 ± 0.80 | 0.944 |
| % EBMI | 27.84 ± 0.76 | 27.76 ± 0.80 | 0.944 |
| **Comorbidities, %** |  |  |  |
| T2D | 8 (28.6%) | 8 (28.6%) | 1.000 |
| Dyslipidemia | 19 (67.9%) | 21 (75.0%) | 0.768 |
| HT | 11 (39.3%) | 13 (46.4%) | 0.787 |
| MS | 15 (53.6%) | 18 (64.3%) | 0.587 |
| Sleep Apnea | 4 (14.3%) | 7 (25.0%) | 0.503 |
| Osteoarthritis | 8 (28.6%) | 9 (32.1%) | 1.000 |
| GERD | 7 (25.0%) | 7 (25.0%) | 1.000 |
|  |  |  |  |

Table S1.2 Weight loss variables during the follow-up.

|  |  | **BPD-DS** | **SADI-S** | **p** |
| --- | --- | --- | --- | --- |
| **6** | BMI, kg/m2 | 35.15 ± 0.90 | 36.75 ± 1.02 | 0.253 |
|  | EBMIL, % | 63.64 ± 2.90 | 58.16 ± 3.09 | 0.209 |
|  | TWL, % | 32.98 ± 1.42 | 30.06 ± 1.46 | 0.163 |
|  | TWL≥20%, n (%) | 20 (100.0%) | 22 (91.70) | 0.493 |
|  | n | 20 | 24 |  |
| **12** | BMI, kg/m2 | 29.77 ± 0.70 | 31.54 ± 1.19 | 0.208 |
|  | EBMIL, % | 82.89 ± 2.47 | 77.82 ± 3.89 | 0.277 |
|  | TWL, % | 43.15 ± 1.21 | 40.33 ± 1.82 | 0.204 |
|  | TWL≥20%, n (%) | 26 (100.0%) | 28 (100.0%) | 1.000 |
|  | n | 26 | 28 |  |
| **24** | BMI, kg/m2 | 28.24 ± 0.53 | 31.76 ± 1.37 | **0.023** |
|  | EBMIL, % | 88.14 ± 1.79 | 77.14 ± 4.58 | **0.032** |
|  | TWL, % | 46.35 ± 0.96 | 40.03 ± 2.23 | **0.014** |
|  | TWL≥20%, n (%) | 21 (100.0%) | 24 (96.0%) | 1.000 |
|  | n | 21 | 25 |  |
| **36** | BMI, kg/m2 | 28.67 ± 0.86 | 34.67 ± 1.72 | **0.004** |
|  | EBMIL, % | 86.74 ± 2.96 | 67.16 ± 5.83 | **0.006** |
|  | TWL, % | 45.12 ± 1.61 | 34.95 ± 2.86 | **0.004** |
|  | TWL≥20%, n (%) | 18 (100.0%) | 17 (81.0%) | 0.110 |
|  | n | 18 | 21 |  |
| **48** | BMI, kg/m2 | 29.24 ± 0.89 | 33.91 ± 1.68 | **0.019** |
|  | EBMIL, % | 85.01 ± 3.07 | 69.17 ± 5.73 | **0.021** |
|  | TWL, % | 44.64 ± 1.66 | 36.03 ± 2.87 | **0.014** |
|  | TWL≥20%, n (%) | 17 (100.0%) | 18 (81.8%) | 0.118 |
|  | n | 17 | 22 |  |
| **60** | BMI, kg/m2 | 30.32 ± 1.06 | 34.75 ± 1.42 | **0.014** |
|  | EBMIL, % | 80.59 ± 3.82 | 66.69 ± 4.37 | **0.021** |
|  | TWL, % | 41.89 ± 2.03 | 35.37 ± 2.35 | **0.041** |
|  | TWL≥20%, n (%) | 24 (96.0%) | 18 (90.0%) | 0.577 |
|  | n | 25 | 20 |  |

Data is represented by mean and standard error of the mean (SEM). Unpaired Student’s t-test or Mann-Whitney U test. Significant differences at bold. BMI, body mass index; TWL, total weight loss; EBMIL, excess body mass index loss; n – number of cases; BPD/DS, biliopancreatic diversion with duodenal switch; SADI-S, single anastomosis duodeno-ileal with sleeve gastrectomy.

Table S1.3 Quality of life questionnaire scores 60 + months after surgery

| **Self Esteem** |  | *Much Worse* | *Worse* | *The Same* | *Better* | *Much Better* |
| --- | --- | --- | --- | --- | --- | --- |
|  | **BPD-DS** | 0.0% | 0.0% | 0.0% | 50.0% | 50.0% |
|  | **SADI-S** | 0.0% | 6.7% | 6.7% | 20.0% | 66.7% |
| **Physical** |  | *Much Less* | *Less* | *The Same* | *More* | *Much More* |
|  | **BPD-DS** | 0.0% | 0.0% | 0.0% | 50.0% | 50.0% |
|  | **SADI-S** | 0.0% | 6.7% | 6.7% | 20.0% | 66.7% |
| **Social** |  | *Much Less* | *Less* | *The Same* | *More* | *Much More* |
|  | **BPD-DS** | 0.0% | 0.0% | 0.0% | 50.0% | 50.0% |
|  | **SADI-S** | 0.0% | 13.3% | 13.3% | 6.7% | 66.7% |
| **Labor** |  | *Much Less* | *Less* | *The Same* | *More* | *Much More* |
|  | **BPD-DS** | 0.0% | 0.0% | 8.3% | 41.7% | 50.0% |
|  | **SADI-S** | 0.0% | 13.3% | 13.3% | 13.3% | 60.0% |
| **Sexual** |  | *Much Less* | *Less* | *The Same* | *More* | *Much More* |
|  | **BPD-DS** | 0.0% | 0.0% | 72.7% | 18.2% | 9.1% |
|  | **SADI-S** | 0.0% | 7.1% | 42.9% | 0.0% | 50.0% |
| **Scoring** |  | *Greatly Diminished* | *Diminished* | *Minimal to no change* | *Improved* | *Greatly Improved* |
|  | **BPD-DS** | 0.0% | 0.0% | 0.0% | 45.5% | 54.5% |
|  | **SADI-S** | 0.0% | 7.1% | 14.3% | 7.1% | 71.4% |
|  | **p** | **0.034** | | | | |

Fisher’s exact test was used to compare the quality-of-life score between the surgical groups. Significant differences at bold. BPD/DS, biliopancreatic diversion with duodenal switch; SADI-S, single anastomosis duodeno-ileal with sleeve gastrectomy.

Table S1.4 Remission of obesity associated medical problems after surgery.

|  | **Pre-operative n** | **Out** |  | **12 months** | | | **24 months** | | | **36 months** | | | **48 months** | | | **60 months** | | | |
| --- | --- | --- | --- | --- | --- | --- | --- | --- | --- | --- | --- | --- | --- | --- | --- | --- | --- | --- | --- |
|  | BPD/DS | SADI-S |  | BPD/DS | SADI-S | p | BPD/DS | SADI-S | p | BPD/DS | SADI-S | p | BPD/DS | SADI-S | p | BPD/DS | SADI-S | p |  |
| **T2D** | 8 | 8 | CR | 6/6(100%) | 4/6(77%) | 0.455 | 4/4(100%) | 5/7(71%) |  | 3/3(100%) | 3/6(50%) | 0.643 | 3/3(100%) | 1/2(50%) | 0.400 | 5/5(100%) | 4/6(67%) | >0.999 |  |
|  |  |  | PR | / | / |  | / | / |  | / | / |  | / | / |  | / | / |  |  |
|  |  |  | I | / | 2/6 (33%) |  |  | 2/7 (29%) | 0.491 | / | 2/6(33%) |  | / | 1/2(50%) |  | / | 1/6(17%) |  |  |
|  |  |  | U | / | / |  | / | / |  | / | 1/6(17%) |  | / | / |  | 1/6(17%) | 1/6(17%) |  |  |
|  |  |  | R | / | / |  | / | / |  | / | / |  | / | / |  | / | / |  |  |
| **HT** | 11 | 13 | CR | 8/10(80%) | 10/13(77%) | >0.999 | 6/7(86%) | 7/10(70%) | 0.603 | 3/4(75%) | 7/11(58%) | >0.999 | 5/5(100%) | 7/12(58%) | 0.245 | 8/11(73%) | 7/12(58%) | 0.667 |  |
| **DL** | 19 | 21 | CR | 7/13(54%) | 5/15(33%) | 0.473 | 8/10(80%) | 5/9(56%) | 0.443 | 3/8(38%) | 8/11(72%) | 0.184 | 2/6(33%) | / | 0.467 | 4/9(44%) | 3/9(33%) | >0.999 |  |
|  |  |  | I | 4/13(31%) | 8/15(53%) |  | 2/10(20%) | 3/9(33%) |  | 4/8(50%) | 1/11(9%) |  | 4/6(67%) | 4/4(100%) |  | 5/9(56%) | 5/9(56%) |  |  |

Pre-operative “n” represents the number of patients with the respective disease before surgery. Out, outcome; CR, complete remission; PR, partial remission; I, improved; U, unchanged; R, recurrence; T2D, type 2 diabetes; HT, hypertension; DL, dyslipidemia; BPD/DS, biliopancreatic diversion with duodenal switch; SADI-S, single anastomosis duodeno-ileal with sleeve gastrectomy. Fisher’s exact test was used to compare the associated medical problems between the surgical groups. No significant differences were observed.

Table S1.5 Laboratory data of patients submitted to biliopancreatic diversion with duodenal switch (BPD/DS) and single anastomosis duodeno-ileal bypass with sleeve gastrectomy (SADI-S) during a follow-up of 60 or more months

|  |  | **0** | **6** | **12** | **24** | **36** | **48** | **60** |
| --- | --- | --- | --- | --- | --- | --- | --- | --- |
| **Glucose, mg/dL** | **BPD-DS** | 116.4 ± 9.4 (n=26) | 87.4 ± 2.1 (n=21) | 84.6 ± 1.6 (n=26) | 84.5 ± 3.3 (n=15) | 86.0 ± 2.4 (n=14) | 85.8 ± 2.8 (n=11) | 85.9 ± 1.5 (n=16) |
|  | **SADI-S** | 118.3 ± 9.3 (n=27) | 89.3 ± 4.6 (n=20) | 83.3 ± 2.3 (n=26) | 83.3 ± 1.8 (n=19) | 83.6 ± 3.2 (n=18) | 87.6 ± 4.4 (n=9) | 84.2 ± 2.7 (n=15) |
|  | **p** | 0.817 | 0.620 | 0.193 | 1.000 | 0.267 | 1.000 | 0.163 |
| **HbA1c, %** | **BPD-DS** | 5.93 ± 0.28 (n=26) | 4.97 ± 0.10 (n=20) | 4.38 ± 0.27 (n=22) | 4.69 ± 0.14 (n=14) | 4.83 ± 0.19 (n=8) | 5.04 ± 0.14 (n=5) | 5.31 ± 0.33 (n=11) |
|  | **SADI-S** | 6.23 ± 0.31 (n=26) | 5.04 ± 0.14 (n=13) | 5.21 ± 0.23 (n=15) | 5.08 ± 0.20 (n=12) | 5.69 ± 0.61 (n=13) | 5.58 ± 0.56 (n=4) | 5.02 ± 0.42 (n=12) |
|  | **p** | 0.735 | 0.957 | **0.045** | 0.347 | 0.414 | 0.556 | 0.880 |
| **Insulin, µUI/mL** | **BPD-DS** | 23.36 ± 2.22 (n=26) | 7.10 ± 0.93 (n=20) | 4.90 ± 0.56 (n=22) | 6.11 ± 1.80 (n=14) | 7.21 ± 1.70 (n=8) | 7.23 ± 1.78 (n=6) | 4.53 ± 0.91 (n=6) |
|  | **SADI-S** | 23.46 ± 3.01 (n=25) | 7.56 ± 1.00  (n=12) | 4.85 ± 0.95 (n=13) | 4.40 ± 0.76 (n=6) | 4.30 ± 0.90 (n=6) | 5.20 ± 1.30 (n=2) | 5.80 ± 2.07 (n=5) |
|  | **p** | 0.785 | 0.632 | 0.775 | 0.602 | 0.491 | 0.643 | 0.792 |
| **Total cholesterol, mg/dL** | **BPD-DS** | 197.15 ± 7.08  (n=26) | 162.05 ± 6.50  (n=20) | 144.50 ± 7.22  (n=22) | 130.00 ± 7.30  (n=14) | 138.27 ± 8.45  (n=11) | 125.86 ± 4.86  (n=7) | 152.15 ± 7.88  (n=13) |
|  | **SADI-S** | 181.15 ± 6.53  (n=26) | 159.32 ± 10.28  (n=19) | 165.55 ± 7.36  (n=20) | 182.23 ± 7.97  (n=13) | 171.23 ± 10.51  (n=13) | 171.50 ± 11.58  (n=6) | 166.77 ± 9.07  (n=13) |
|  | **p** | 0.126 | 0.728 | **0.025** | **<0.001** | **0.030** | **0.005** | 0.101 |
| **Triglycerides, mg/dL** | **BPD-DS** | 135.81 ± 10.93  (n=26) | 90.20 ± 6.73  (n=20) | 76.00 ± 5.08  (n=22) | 57.29 ± 4.70  (n=14) | 75.36 ± 10.25  (n=11) | 67.43 ± 11.28  (n=7) | 84.62 ± 8.52  (n=13) |
|  | **SADI-S** | 138.50 ± 14.56  (n=26) | 90.83 ± 8.39   (n=18) | 81.30 ± 7.37  (n=20) | 75.92 ± 8.93  (n=13) | 85.08 ± 16.98  (n=13) | 79.00 ± 8.99  (n=7) | 82.86 ± 12.75  (n=14) |
|  | **p** | 0.721 | 0.828 | 0.597 | 0.202 | 0.776 | 0.318 | 0.720 |
| **HDL, mg/dL** | **BPD-DS** | 52.65 ± 2.95  (n=26) | 49.90 ± 2.81  (n=20) | 50.68 ± 2.57  (n=22) | 54.50 ± 4.31  (n=14) | 50.64 ± 3.10  (n=11) | 45.00 ± 1.00  (n=7) | 51.15 ± 2.74  (n=13) |
|  | **SADI-S** | 46.65 ± 2.24  (n=26) | 46.26 ± 4.72   (n=19) | 55.05 ± 5.38  (n=20) | 69.62 ± 5.41  (n=13) | 65.23 ± 3.45  (n=13) | 71.33 ± 14.21  (n=6) | 60.15 ± 5.97  (n=13) |
|  | **p** | 0.181 | 0.134 | 0.840 | **0.019** | **0.002** | 0.073 | 0.311 |
| **B12 vitamin, pg/mL** | **BPD-DS** | 374.15 ± 27.77  (n=26) | 490.10 ± 48.18   (n=21) | 471.69 ± 47.13   (n=26) | 339.47 ± 27.59   (n=15) | 420.86 ± 51.34  (n=14) | 653.75 ± 137.50  (n=12) | 703.62 ± 84.95  (n=13) |
|  | **SADI-S** | 400.15 ± 29.72  (n=20) | 472.62 ± 39.94   (n=21) | 521.36 ± 61.37  (n=25) | 540.48 ± 89.98   (n=21) | 464.56 ± 63.43   (n=18) | 424.56 ± 67.75  (n=9) | 539.27 ± 73.27   (n=11) |
|  | **p** | 0.394 | 0.831 | 0.611 | 0.160 | 0.722 | 0.193 | 0.167 |
| **25-OH-D vitamin, ng/mL** | **BPD-DS** | 7.50 ± 0.00  (n=1) | 18.27 ± 4.40  (n=6) | 13.84 ± 2.37  (n=13) | 13.64 ± 2.45   (n=9) | 16.42 ± 3.20   (n=9) | 12.93 ± 2.74   (n=11) | 20.54 ± 3.38   (n=13) |
|  | **SADI-S** | (n=0) | 17.75 ± 2.84  (n=13) | 20.63 ± 2.06 (n=18) | 18.93 ± 1.92  (n=19) | 18.76 ± 1.97  (n=16) | 27.82 ± 4.60  (n=9) | 21.58 ± 3.09  (n=11) |
|  | **p** | - | 0.898 | 0.056 | 0.095 | 0.598 | **0.016** | 0.733 |
| **PTH, pmol/L** | **BPD-DS** | 10.14 ± 1.92  (n=9) | 9.86 ± 3.03  (n=6) | 9.28 ± 2.02  (n=8) | 12.46 ± 4.60   (n=6) | 26.95 ± 12.22  (n=8) | 19.64 ± 4.21  (n=7) | 30.80 ± 6.35   (n=3) |
|  | **SADI-S** | 8.18 ± 0.76  (n=11) | 6.64 ± 0.99   (n=4) | 10.00 ± 2.06  (n=7) | 10.68 ± 1.27  (n=7) | 14.20 ± 1.40  (n=10) | 9.51 ± 1.06  (n=6) | 48.58 ± 34.69  (n=4) |
|  | **p** | 0.656 | 0.476 | 0.536 | 0.539 | 0.897 | 0.101 | 0.629 |
| **Calcium, mg/dL** | **BPD-DS** | 9.28 ± 0.11   (n=9) | 9.50 ± 0.23   (n=6) | 8.97 ± 0.14   (n=11) | 8.86 ± 0.10  (n=8) | 8.85 ± 0.18   (n=11) | 9.04 ± 0.19  (n=11) | 9.03 ± 0.14   (n=12) |
|  | **SADI-S** | 9.42 ± 0.10  (n=18) | 9.38 ± 0.10   (n=12) | 9.12 ± 0.12   (n=15) | 9.06 ± 0.10   (n=16) | 9.05 ± 0.10   (n=16) | 9.21 ± 0.13   (n=8) | 9.17 ± 0.13  (n=11) |
|  | **p** | 0.596 | 1.000 | 0.683 | 0.320 | 0.577 | 0.600 | 0.487 |
| **Hemoglobin, g/dL** | **BPD-DS** | 14.18 ± 0.28 (n=27) | 13.27 ± 0.23 (n=21) | 12.78 ± 0.22 (n=25) | 12.89 ± 0.34  (n=15) | 12.59 ± 0.35 (n=15) | 12.65 ± 0.32 (n=11) | 12.67 ± 0.32 (n=19) |
|  | **SADI-S** | 14.27 ± 0.31 (n=27) | 13.25 ± 0.23 (n=20) | 13.16 ± 0.27 (n=26) | 12.98 ± 0.37  (n=21) | 13.16 ± 0.33 (n=21) | 13.08 ± 0.38 (n=12) | 13.14 ± 0.41 (n=17) |
|  | **p** | 0.931 | 0.764 | 0.375 | 0.950 | 0.309 | 0.487 | 0.315 |
| **Total proteins, g/dL** | **BPD-DS** | 7.22 ± 0.09 (n=25) | 6.59 ± 0.09 (n=21) | 6.57 ± 0.09  (n=26) | 6.69 ± 0.12  (n=15) | 6.73 ± 0.09  (n=15) | 6.76 ± 0.15  (n=12) | 6.82 ± 0.13  (n=11) |
|  | **SADI-S** | 7.21 ± 0.07 (n=26) | 6.76 ± 0.11  (n=19) | 6.72 ± 0.08  (n=25) | 6.82 ± 0.07  (n=19) | 6.71 ± 0.15  (n=17) | 6.81 ± 0.14  (n=8) | 6.70 ± 0.15  (n=11) |
|  | **p** | 0.865 | 0.258 | 0.195 | 0.157 | 0.737 | 0.851 | 0.699 |
| **Iron, µg/dL** | **BPD-DS** | 88.50 ± 5.07 (n=26) | 64.86 ± 3.45 (n=21) | 76.54 ± 5.11 (n=26) | 72.20 ± 7.41  (n=15) | 63.38 ± 5.28 (n=13) | 70.17 ± 5.71 (n=12) | 78.00 ± 9.51 (n=13) |
|  | **SADI-S** | 84.95 ± 7.15 (n=20) | 73.20 ± 5.56  (n=20) | 78.88 ± 5.54 (n=25) | 95.86 ± 8.05  (n=21) | 86.12 ± 9.96 (n=17) | 101.30 ± 14.42 (n=10) | 93.00 ± 16.26 (n=10) |
|  | **p** | 0.358 | 0.080 | 0.445 | **0.042** | 0.079 | 0.093 | 0.684 |
| **Ferritin, ng/dL** | **BPD-DS** | 71.85 ± 43.47 (n=25) | 209.19 ± 35.12 (n=21) | 192.04 ± 28.92 (n=23) | 126.09 ± 36.15 (n=12) | 59.48 ± 21.76 (n=10) | 123.14 ± 29.55 (n=9) | 70.61 ± 20.74 (n=13) |
|  | **SADI-S** | 172.94 ± 34.62 (n=19) | 204.34 ± 36.36  (n=20) | 187.11 ± 40.24 (n=23) | 142.87 ± 31.96 (n=17) | 98.85 ± 28.45 (n=17) | 46.00 ± 10.47 (n=10) | 50.25 ± 15.32 (n=11) |
|  | **p** | 0.678 | 0.917 | 0.435 | 0.711 | 0.604 | **0.035** | 0.589 |

Data is represented by mean and standard error of the mean (SEM).Unpaired Student’s t-test or Mann-Whitney U test. Significant differences at bold.

Table S1.6. Comparison of nutritional deficiencies of patients submitted to submitted to biliopancreatic diversion with duodenal switch (BPD/DS) and single anastomosis duodeno-ileal bypass with sleeve gastrectomy (SADI-S) during a follow-up of 60 or more months

|  | |  | | **6** | **12** | **24** | **36** | **48** | **60** |
| --- | --- | --- | --- | --- | --- | --- | --- | --- | --- |
| **B12 vitamin, n (%)** | | **BPD-DS** | 0 (0.0%) (21) | 0 (0.0%) (26) | 1 (6.7%) (15) | 1 (7.1%) (14) | 0 (0.0%) (12) | 0 (0.0%) (13) |  |
|  |  | **SADI-S** | 0 (0.0%) (21) | 1 (4.0%) (25) | 0 (0.0%) (21) | 1 (5.6%) (18) | 0 (0.0%) (9) | 1 (9.1%) (11) |  |
|  |  | **p** | - | 0.490 | 0.417 | 1.00. | - | 0.458 |  |
| **25-OH-D vitamin, n (%)** | | **BPD-DS** | 1 (16.7%) (6) | 6 (46.2%) (13) | 2 (22.2%) (9) | 3 (33.3%) (9) | **5 (45.5%) (11)** | 3 (23.1%) (13) |  |
|  |  | **SADI-S** | 2 (15.4%) (13) | 4 (22.2%) (25) | 3 (15.8%) (19) | 3 (18.8%) (16) | **0 (0.0%) (9)** | 1 (9.1%) (11) |  |
|  |  | **p** | 1.000 | 0.247 | 1.000 | 0.630 | **0.038** | 0.596 |  |
| **Total proteins, n (%)** | | **BPD-DS** | 9 (42.9%) (21) | 9 (34.6%) (26) | 6 (40.0%) (15) | 5 (33.3)  (n=15) | 5 (41.7)  (n=12) | 3 (27.3)  (n=11) |  |
|  |  | **SADI-S** | 5 (26.3%) (19) | 8 (32.0%) (25) | 2 (10.5%) (19) | 4 (23.5)  (n=17) | 2 (25.0)  (n=8) | 2 (18.2)  (n=11) |  |
|  |  | **p** | 0.333 | 1.000 | 0.100 | 0.699 | 0.642 | 1.000 |  |
| **Iron, µg/dL** | | **BPD-DS** | 3 (14.3)  (n=21) | 4 (15.4)  (n=26) | 4 (26.7)  (n=15) | 3 (23.1)  (n=13) | 1 (8.3)  (n=12) | 3 (23.1)  (n=13) |  |
|  |  | **SADI-S** | 4 (20.0)  (n=20) | 4 (16.0)  (n=25) | 2 (9.5)  (n=21) | 4 (23.5)  (n=17) | 1 (10.0)  (n=10) | 1 (10.0)  (n=10) |  |
|  |  | **p** | 0.697 | 1.000 | 0.210 | 1.000 | 1.000 | 0.604 |  |

Fisher’s exact test was used to compare the number of patients with nutritional deficiencies between the surgical groups. Significant differences at bold.
