# Supplementary material for: Long-Term Outcomes of Single and Dual Anastomosis Duodenal Switch
Source: Obes Surg. 2025 Aug 9;35(9):3791–800. doi: 10.1007/s11695-025-08114-x (PMC12457490; doi:10.1007/s11695-025-08114-x)
Supplement: Supplementary file 6 — DOCX (16.8 KB) [file 11695_2025_8114_MOESM4_ESM.docx]

|  | 6 | 12 | 24 | 36 | 48 | 60 |
| --- | --- | --- | --- | --- | --- | --- |
| Sex | -2.363 (-5.323;0.596)  p=0.116 | **-4.296 (-7.636; -0.957)**  **p=0.012** | **-5.270 (-9.195; -1.344)**  **p=0.009** | **-7.577 (-12.339; -2.815)**  **p=0.002** | **-7.718 (-12.330; -3.106)**  **p=0.001** | -4.978 (-10.604; 0.649)  p=0.082) |
| Age at surgery | 0.003 (-0.109; 0.114)  p=0.960 | **-0.167 (-0.295; -0.040)**  **p=0.011** | -0.127 (-0.281; 0.026)  p=0.101 | -0.136 (-0.316; 0.045)  p=0.139 | -0.057 (-0.242; 0.128)  p=0.540 | -0.107 (-0.331; 0.118)  p=0.348 |
| BMI at surgery | -0.241 (-0.494; 0.012)  p=0.062 | 0.133 (-0.177; 0.442)  p=0.397 | 0.287 (-0.086; 0.660)  p=0.130 | 0.352 (-0.041; 0.746)  p=0.079 | **0.575 (0.156; 0.993)**  **p=0.008** | **0.639 (0.150; 1.128)**  **p=0.011)** |
| T2D at surgery | -0.040 (-3.150; 3.070)  p=0.980 | -1.760 (-5.337; 1.816)  p=0.331 | -0.090 (-4.343; 4.183)  p=0.970 | -1.580 (-6.786; 3.627)  p=0.548 | -0.999 (-6.112; 4.114) p=0.699 | 1.040 (-4.586; 6.666)  p=0.714 |

Supplementary Table 1 – Impact of participants sex, age, BMI, and presence of T2D before surgery on the % of total weight loss after surgery

Linear regression analysis. Significant differences at bold. BMI- Body mass index, T2D – type 2 diabetes
