# Supplementary material for: Long-Term Outcomes of Single and Dual Anastomosis Duodenal Switch
Source: Obes Surg. 2025 Aug 9;35(9):3791–800. doi: 10.1007/s11695-025-08114-x (PMC12457490; doi:10.1007/s11695-025-08114-x)
Supplement: Supplementary file 7 — DOCX (20.2 KB) [file 11695_2025_8114_MOESM5_ESM.docx]

Supplementary Table 2. Weight loss variables during the follow-up.

|  |  | **BPD-DS** | | **SADI-S** | |  | **p** |
| --- | --- | --- | --- | --- | --- | --- | --- |
|  |  | **Observed Mean**  **(M ± SEM)** | **Adjusted Mean ^1^**  **(M [95% CI])** | **Observed Mean**  **(M ± SEM)** |  | **Adjusted Mean ^1^**  **(M [95% CI])** |  |
| **6** | BMI, kg/m2 | 36.14 ± 1.31 | 34.56 [33.22; 35.91] | 35.27 ± 0.60 |  | 35.76 [35.03; 36.50] | 0.128 |
|  | EBMIL, % | 62.03 ± 3.20 | 65.00 [59.88; 70.12] | 61.65 ± 1.59 |  | 60.72 [57.92; 63.53] | 0.154 |
|  | TWL, % | 32.32 ± 1.51 | 32.97 [30.34; 35.61] | 30.74 ± 0.70 |  | 30.58 [29.14; 32.03] | 0.123 |
|  | TWL≥20%, n (%) | 20 (95.2%) | | 65 (97.5%) | | |  |
|  | n | 21 | | 67 | | |  |
| **12** | BMI, kg/m2 | 29.89 ± 0.68 | 28.78 [27.31; 30.26] | 29.91 ± 0.57 |  | 30.29 [29.44; 31.13] | 0.086 |
|  | EBMIL, % | 82.90 ± 2.38 | 85.50 [79.86; 91.15] | 81.81 ± 1.91 |  | 80.93 [77.70; 84.15] | 0.171 |
|  | TWL, % | 29.89 ± 0.68 | 43.44 [40.60; 46.28] | 29.91 ± 0.56 |  | 40.75 [39.13; 42.37] | 0.111 |
|  | TWL≥20%, n (%) | 27 (100.0%) | | 80 (100.0%) | | |  |
|  | n | 27 | | 80 | | |  |
| **24** | BMI, kg/m2 | 28.24 ± 0.53 | 27.42 [25.51; 29.33] | 29.90 ± 0.60 |  | 30.12 [29.15; 31.08] | **0.015** |
|  | EBMIL, % | 88.14 ± 1.79 | 86.68 [82.44; 96.92] | 81.91 ± 2.14 |  | 81.50 [77.85; 85.15] | 0.051 |
|  | TWL, % | 46.35 ± 0.96 | 45.76 [42.12; 49.40] | 40.93 ± 1.04 |  | 41.09 [39.25; 42.93] | **0.027** |
|  | TWL≥20%, n (%) | 21 (100.0%) | | 21 (100.0%) | | |  |
|  | n | 21 | | 79 | | |  |
| **36** | BMI, kg/m2 | 28.89 ± 0.84 | 27.89 [25.65; 30.14] | 31.15 ± 0.68 |  | 31.41 [30.29; 32.52] | **0.007** |
|  | EBMIL, % | 86.51 ± 2.81 | 88.65 [80.27; 97.03] | 77.13 ± 2.40 |  | 76.58 [72.43; 80.74] | **0.013** |
|  | TWL, % | 45.52 ± 1.58 | 45.07 [40.83; 49.33] | 38.47 ± 1.18 |  | 38.59 [36.48; 40.70] | **0.009** |
|  | TWL≥20%, n (%) | 19 (100.0%) | | 68 (91.9%) | | |  |
|  | n | 19 | | 74 | | |  |
| **48** | BMI, kg/m2 | 29.39 ± 0.85 | 28.73 [26.39; 30.35] | 31.34 ± 0.67 |  | 31.51 [30.35; 32.66] | **0.040** |
|  | EBMIL, % | 85.02 ± 2.89 | 85.58 [76.73; 94.42] | 75.65 ± 2.45 |  | 75.51 [71.16; 79.86] | **0.048** |
|  | TWL, % | 45.19 ± 1.66 | 43.78 [39.32; 48.24] | 38.01 ± 1.24 |  | 38.37 [36.18; 40.57] | **0.036** |
|  | TWL≥20%, n (%) | 18 (100.0%) | | 65 (91.5%) | | |  |
|  | n | 18 | | 71 | | |  |
| **60+** | BMI, kg/m2 | 30.32 ± 1.06 | 30.05 [27.75; 32.35] | 32.69 ± 0.74 |  | 32.79 [31.40; 34.18] | **0.048** |
|  | EBMIL, % | 80.59 ± 3.82 | 80.64 [71.87; 89.41] | 70.51 ± 2.76 |  | 70.49 [65.18; 75.80] | 0.054 |
|  | TWL, % | 41.89 ± 2.03 | 41.23 [36.82; 45.64] | 35.55 ± 1.42 |  | 35.80 [33.13; 38.46] | **0.040** |
|  | TWL≥20%, n (%) | 24 (96.0%) | | 61 (91.0%) | | |  |
|  | n | 25 | | 67 | | |  |

^1^ Adjusted means are estimated marginal means derived from ANCOVA, controlling for baseline BMI, age, sex, and presence of type 2 diabetes. p-values correspond to between-group comparisons adjusted for covariates. Significant differences at bold.

BMI, body mass index; TWL, total weight loss; EBMIL, excess body mass index loss; n – number of cases; BPD/DS, biliopancreatic diversion with duodenal switch; SADI-S, single anastomosis duodeno-ileal with sleeve gastrectomy.
