# Supplementary material for: Long-Term Outcomes of Single and Dual Anastomosis Duodenal Switch
Source: Obes Surg. 2025 Aug 9;35(9):3791–800. doi: 10.1007/s11695-025-08114-x (PMC12457490; doi:10.1007/s11695-025-08114-x)
Supplement: Supplementary file 8 — DOCX (20.9 KB) [file 11695_2025_8114_MOESM6_ESM.docx]

Supplementary Table 3. Remission of obesity associated medical problems after surgery.

|  | **Pre-operative n** | **Out** |  | **12 months** | | | **24 months** | | | **36 months** | | | **48 months** | | | **60 months** | | | |
| --- | --- | --- | --- | --- | --- | --- | --- | --- | --- | --- | --- | --- | --- | --- | --- | --- | --- | --- | --- |
|  | BPD/DS | SADI-S |  | BPD/DS | SADI-S | p | BPD/DS | SADI-S | p | BPD/DS | SADI-S | p | BPD/DS | SADI-S | p | BPD/DS | SADI-S | p |  |
| **T2D** | 8 | 16 | CR | 6/6(100%) | 7/10(70%) | 0.679 | 4/4(100%) | 10/13(77%) |  | 3/3(100%) | 7/11(64%) | 0.629 | 3/3(100%) | 5/6(83%) | >0.999 | 5/6(83%) | 7/9(78%) | >0.999 |  |
|  |  |  | PR | / | / |  | / | / |  | / | / |  | / | / |  | / | / |  |  |
|  |  |  | I | / | 2/10 (20%) |  |  | 2/13 (15%) | >0.999 | / | 3/11(27%) |  | / | 1/6(17%) |  | / | 1/9(11%) |  |  |
|  |  |  | U | / | 1/10 (10%) |  | / | 1/13 (8%) |  | / | 1/11(9%) |  | / | / |  | 1/6(17%) | 1/9(11%) |  |  |
|  |  |  | R | / | / |  | / | / |  | / | / |  | / | / |  | / | / |  |  |
| **HT** | 12 | 38 | CR | 9/11 (82%) | 25/34(74%) | 0.705 | 7/8(88%) | 22/33(67%) | 0.398 | 4/5(80%) | 23/31(74%) | >0.999 | 5/5(100%) | 24/34(71%) | 0.302 | 8/11(73%) | 24/33(73%) | >0.999 |  |
| **DL** | 19 | 51 | CR | 7/13(54%) | 14/34(41%) | 0.762 | 8/10(80%) | 9/22(41%) | 0.080 | 3/8(38%) | 11/20(55%) | 0.096 | 2/6(33%) | 5/11(46%) | 0.493 | 4/9(44%) | 6/21(29%) | 0.405 |  |
|  |  |  | I | 4/13(31%) | 13/34(38%) |  | 2/10(20%) | 6/22(27%) |  | 4/8(50%) | 2/20(10%) |  | 4/6(67%) | 4/11(36%) |  | 5/9(56%) | 11/21(52%) |  |  |
| **MS** | 15 | 43 | CR | 11/13(85%) | 33/38(87%) | >0.999 | 8/8(100%) | 31/36(86%) | 0.566 | 9/10(90%) | 27/31(87%) | 0.644 | 6/6(100%) | 28/33(85%) | 0.574 | 12/13(92%) | 21/27(78%) | 0.393 |  |

Pre-operative “n” represents the number of patients with the respective disease before surgery. Out, outcome; CR, complete remission; PR, partial remission; I, improved; U, unchanged; R, recurrence; T2D, type 2 diabetes; HT, hypertension; DL, dyslipidemia; MS, metabolic syndrome; BPD/DS, biliopancreatic diversion with duodenal switch; SADI-S, single anastomosis duodeno-ileal with sleeve gastrectomy. Fisher’s exact test was used to compare the associated medical problems between the surgical groups. No significant differences were observed.
