# Supplementary material for: Long-Term Outcomes of Single and Dual Anastomosis Duodenal Switch
Source: Obes Surg. 2025 Aug 9;35(9):3791–800. doi: 10.1007/s11695-025-08114-x (PMC12457490; doi:10.1007/s11695-025-08114-x)
Supplement: Supplementary file 9 — DOCX (34.3 KB) [file 11695_2025_8114_MOESM7_ESM.docx]

Supplementary Table 4. Laboratory data of patients submitted to biliopancreatic diversion with duodenal switch (BPD/DS) and single anastomosis duodeno-ileal bypass with sleeve gastrectomy (SADI-S) during a follow-up of 60 or more months

|  |  | **0** | **6** | **12** | **24** | **36** | **48** | **60** |
| --- | --- | --- | --- | --- | --- | --- | --- | --- |
| **Glucose, mg/dL** | **BPD-DS** | 115.30 ± 9.12  (n=27) | 87.38 ± 2.12  (n=21) | 84.37 ± 1.53  (n=27) | 84.56 ± 3.04  (n=16) | 86.00 ± 2.36  (n=14) | 85.82 ± 2.75  (n=11) | 85.94 ± 1.49  (n=16) |
|  | **SADI-S** | 106.17 ± 3.62  (n=82) | 84.76 ± 1.89  (n=54) | 81.62 ± 1.01  (n=77) | 82.45 ± 0.88  (n=64) | 81.74 ± 1.22  (n=58) | 82.57 ± 1.50  (n=37) | 84.13 ± 1.67  (n=47) |
|  | **p** | 0.392 | 0.088 | 0.054 | 0.763 | 0.073 | 0.215 | 0.131 |
| **HbA1c, %** | **BPD-DS** | 5.90 ± 0.28  (n=27) | 4.97 ± 0.10  (n=20) | **4.36 ± 0.26**  **(n=23)** | 4.65 ± 0.14  (n=15) | 4.83 ± 0.19  (n=8) | 5.04 ± 0.14  (n=5) | 5.31 ± 0.33  (n=11) |
|  | **SADI-S** | 5.88 ± 0.13  (n=77) | 5.03 ± 0.08  (n=30) | **5.11 ± 0.10**  **(n=37)** | 5.01 ± 0.09  (n=31) | 5.31 ± 0.29  (n=28) | 5.30 ± 0.27  (n=9) | 5.28 ± 0.21  (n=27) |
|  | **p** | 0.997 | 0.720 | **0.002** | 0.083 | 0.320 | 0.606 | 0.465 |
| **Insulin, µUI/mL** | **BPD-DS** | 23.41 ± 2.14  (n=27) | 7.10 ± 0.93  (n=20) | 4.93 ± 0.54  (n=23) | 6.11 ± 1.80  (n=14) | 7.21 ± 1.70  (n=8) | 7.23 ± 1.78  (n=6) | 4.53 ± 0.91  (n=6) |
|  | **SADI-S** | 21.78 ± 1.39  (n=76) | 7.39 ± 0.85  (n=28) | 5.17 ± 0.50  (n=29) | 4.69 ± 0.50  (n=14) | 5.07 ± 0.77  (n=11) | 4.72 ± 0.75  (n=5) | 5.68 ± 0.91  (n=13) |
|  | **p** | 0.399 | 0.917 | 0.625 | 0.541 | 0.717 | 0.329 | 0.521 |
| **Total cholesterol, mg/dL** | **BPD-DS** | 195.81 ± 6.95  (n=27) | 162.05 ± 6.50  (n=20) | **142.87 ± 7.09**  **(n=23)** | **129.47 ± 6.82**  **(n=15)** | **138.27 ± 8.45**  **(n=11)** | **125.86 ± 4.86**  **(n=7)** | **152.15 ± 7.88**  **(n=13)** |
|  | **SADI-S** | 187.47 ± 3.92  (n=81) | 162.55 ± 5.56  (n=49) | **166.64 ± 4.08**  **(n=56)** | **169.76 ± 5.33**  **(n=42)** | **174.47 ± 6.04**  **(n=34)** | **167.00 ± 6.11**  **(n=19)** | **174.72 ± 4.82**  **(n=40)** |
|  | **p** | 0.340 | 0.968 | **0.003** | **<0.001** | **0.003** | **<0.001** | **0.008** |
| **Triglycerides, mg/dL** | **BPD-DS** | 133.37 ± 10.79  (n=27) | 90.20 ± 6.73  (n=20) | 76.35 ± 4.86  (n=23) | **57.07 ± 4.38**  **(n=15)** | 75.36 ± 10.25  (n=11) | 67.43 ± 11.28  (n=7) | 84.62 ± 8.52  (n=13) |
|  | **SADI-S** | 128.24 ± 6.85  (n=80) | 88.73 ± 4.37  (n=48) | 77.14 ± 3.59  (n=57) | **78.46 ± 6.57**  **(n=41)** | 83.24 ± 8.02  (n=33) | 76.47 ± 7.25  (n=20) | 83.60 ± 7.32  (n=42) |
|  | **p** | 0.559 | 0.925 | 0.958 | **0.044** | 0.810 | 0.498 | 0.526 |
| **HDL, mg/dL** | **BPD-DS** | 52.19 ± 2.87  (n=27) | **49.90 ± 2.81**  **(n=20)** | 50.04 ± 2.54  (n=23) | 54.00 ± 4.04  (n=15) | **50.64 ± 3.10**  **(n=11)** | **45.00 ± 1.00**  **(n=7)** | 51.15 ± 2.74  (n=13) |
|  | **SADI-S** | 50.90 ± 1.79  (n=81) | **42.84 ± 2.08**  **(n=49)** | 52.89 ± 2.23  (n=57) | 63.92 ± 3.07  (n=40) | **63.21 ± 2.94**  **(n=34)** | **61.89 ± 4.87**  **(n=19)** | 58.22 ± 2.48  (n=40) |
|  | **p** | 0.707 | **0.019** | 0.506 | 0.059 | **0.015** | **0.015** | 0.133 |
| **B12 vitamin, pg/mL** | **BPD-DS** | 379.22 ± 27.20  (n=27) | 490.10 ± 48.18  (n=21) | 467.52 ± 45.54  (n=27) | **335.81 ± 26.06**  **(n=16)** | 420.86 ± 51.34  (n=14) | 653.75 ± 137.50  (n=12) | 703.62 ± 84.95  (n=13) |
|  | **SADI-S** | 384.75 ± 16.19  (n=60) | 558.19 ± 44.23  (n=54) | 568.88 ± 43.60  (n=75) | **583.25 ± 47.67**  **(n=65)** | 518.98 ± 41.99  (n=60) | 550.35 ± 40.86  (n=34) | 580.89 ± 44.09  (n=45) |
|  | **p** | 0.643 | 0.571 | 0.302 | **0.008** | 0.427 | >0.999 | 0.126 |
| **25-OH-D vitamin, ng/mL** | **BPD-DS** | 7.50 ± 0.00  (n=1) | 18.27 ± 4.40  (n=6) | **13.84 ± 2.37**  **(n=13)** | **13.64 ± 2.45**  **(n=9)** | 16.42 ± 3.20  (n=9) | **12.93 ± 2.74**  **(n=11)** | 20.54 ± 3.38  (n=13) |
|  | **SADI-S** | 59.84 ± 45.09  (n=7) | 20.80 ± 1.75  (n=40) | **23.94 ± 1.86**  **(n=58)** | **23.43 ± 1.43**  **(n=59)** | 23.64 ± 1.83  (n=56) | **24.48 ± 2.42**  **(n=31)** | 24.03 ± 1.64  (n=35) |
|  | **p** | 0.250 | 0.667 | **0.006** | **0.007** | 0.152 | **0.011** | 0.479 |
| **PTH, pmol/L** | **BPD-DS** | 9.61 ± 1.79  (n=10) | 9.86 ± 3.03  (n=6) | 8.99 ± 1.81  (n=9) | 12.00 ± 3.92  (n=7) | 26.95 ± 12.22  (n=8) | 19.64 ± 4.21  (n=7) | 30.80 ± 6.35  (n=3) |
|  | **SADI-S** | 8.97 ± 0.49  (n=32) | 8.63 ± 1.16  (n=11) | 8.40 ± 0.64  (n=30) | 9.07 ± 0.53  (n=29) | 11.65 ± 0.89  (n=25) | 10.93 ± 0.94  (n=17) | 23.52 ± 8.87  (n=17) |
|  | **p** | 0.850 | 0.961 | 0.756 | 0.397 | 0.397 | 0.087 | 0.093 |
| **Calcium, mg/dL** | **BPD-DS** | 9.33 ± 0.11  (n=10) | 9.50 ± 0.23  (n=6) | 8.99 ± 0.13  (n=12) | 8.90 ± 0.10  (n=9) | 8.85 ± 0.18  (n=11) | 9.04 ± 0.19  (n=11) | 9.03 ± 0.14  (n=12) |
|  | **SADI-S** | 9.36 ± 0.05  (n=54) | 9.25 ± 0.09  (n=37) | 9.04 ± 0.06  (n=55) | 9.00 ± 0.05  (n=59) | 8.97 ± 0.06  (n=55) | 9.08 ± 0.09  (n=31) | 9.16 ± 0.07  (n=37) |
|  | **p** | 0.985 | 0.504 | 0.876 | 0.543 | 0.796 | 0.888 | 0.484 |
| **Hemoglobin, g/dL** | **BPD-DS** | 14.16 ± 0.27  (n=28) | 13.27 ± 0.23  (n=21) | 12.75 ± 0.21  (n=26) | 12.89 ± 0.32  (n=16) | 12.59 ± 0.35  (n=15) | 12.65 ± 0.32  (n=11) | 12.67 ± 0.32  (n=19) |
|  | **SADI-S** | 14.17 ± 0.15  (n=82) | 13.26 ± 0.20  (n=54) | 13.14 ± 0.16  (n=78) | 12.92 ± 0.21  (n=70) | 13.07 ± 0.18  (n=65) | 13.08 ± 0.23  (n=38) | 13.08 ± 0.23  (n=50) |
|  | **p** | 0.799 | 0.759 | 0.096 | 0.669 | 0.227 | 0.343 | 0.188 |
| **Total proteins, g/dL** | **BPD-DS** | 7.22 ± 0.09  (n=26) | 6.59 ± 0.09  (n=21) | 6.56 ± 0.09  (n=27) | 6.69 ± 0.12  (n=16) | 6.73 ± 0.09  (n=15) | 6.76 ± 0.15  (n=12) | 6.82 ± 0.13  (n=11) |
|  | **SADI-S** | 7.14 ± 0.05  (n=81) | 6.65 ± 0.07  (n=53) | 6.65 ± 0.05  (n=74) | 6.71 ± 0.06  (n=63) | 6.72 ± 0.07  (n=57) | 6.73 ± 0.07  (n=31) | 8.33 ± 1.68  (n=36) |
|  | **p** | 0.471 | 0.407 | 0.263 | 0.561 | 0.978 | >0.999 | 0.464 |
| **Iron, µg/dL** | **BPD-DS** | 86.78 ± 5.17  (n=27) | 64.86 ± 3.45  (n=21) | **75.44 ± 5.03**  **(n=27)** | 71.13 ± 7.01  (n=16) | **63.38 ± 5.28**  **(n=13)** | 70.17 ± 5.71  (n=12) | 78.00 ± 9.51  (n=13) |
|  | **SADI-S** | 82.84 ± 3.64  (n=61) | 70.70 ± 3.69  (n=53) | **86.01 ± 3.45**  **(n=75)** | 90.47 ± 4.59  (n=68) | **92.56 ± 5.01**  **(n=59)** | 91.19 ± 6.79  (n=37) | 96.76 ± 8.81  (n=38) |
|  | **p** | 0.385 | 0.446 | **0.030** | 0.054 | **0.004** | 0.106 | 0.424 |
| **Ferritin, ng/dL** | **BPD-DS** | 169.35 ± 41.84  (n=26) | 209.19 ± 35.12  (n=21) | 188.05 ± 27.97  (n=24) | 123.30 ± 33.37  (n=13) | 59.48 ± 21.76  (n=10) | 123.14 ± 29.55  (n=9) | 70.61 ± 20.74  (n=13) |
|  | **SADI-S** | 192.48 ± 24.28  (n=58) | 200.30 ± 20.72  (n=53) | 187.59 ± 19.19  (n=73) | 160.63 ± 26.61  (n=63) | 137.34 ± 28.08  (n=58) | 113.40 ± 42.47  (n=33) | 74.73 ± 13.40  (n=41) |
|  | **p** | 0.416 | 0.834 | 0.688 | 0.746 | 0.283 | 0.098 | 0.895 |

Data is represented by mean and standard error of the mean (SEM).Unpaired Student’s t-test or Mann-Whitney U test. Significant differences at bold. PTH, parathormone.
