# Supplementary material for: Long-Term Outcomes of Single and Dual Anastomosis Duodenal Switch
Source: Obes Surg. 2025 Aug 9;35(9):3791–800. doi: 10.1007/s11695-025-08114-x (PMC12457490; doi:10.1007/s11695-025-08114-x)
Supplement: Supplementary file 10 — DOCX (20.4 KB) [file 11695_2025_8114_MOESM8_ESM.docx]

|  | |  | | **6** | **12** | **24** | **36** | **48** | **60** |
| --- | --- | --- | --- | --- | --- | --- | --- | --- | --- |
| **B12 vitamin, n (%)** | | **BPD-DS** | 0 (0.0)  (n=21) | 0 (0.0)  (n=27) | 1 (6.3)  (n=16) | 1 (7.1)  (n=14) | 0 (0.0)  (n=12) | 0 (0.0)  (n=13) |  |
|  |  | **SADI-S** | 0 (0.0)  (n=54) | 4 (5.3)  (n=75) | 1 (1.5)  (n=65) | 1 (5.0)  (n=60) | 0 (0.0)  (n=34) | 1 (2.2)  (n=45) |  |
|  |  | **p** | - | 0.571 | 0.358 | 0.576 | - | >0.999 |  |
| **25-OH-D vitamin, n (%)** | | **BPD-DS** | 1 (16.7)  (n=6) | 6 (46.2)  (n=13) | 2 (22.2)  (n=9) | 3 (33.3)  (n=9) | 5 (45.5)  (n=11) | 3 (23.1)  (n=13) |  |
|  |  | **SADI-S** | 7 (17.5)  (n=40) | 10 (17.2)  (n=58) | 6 (10.2)  (n=59) | 7 (12.5)  (n=56) | 5 (16.1)  (n=31) | 4 (11.4)  (n=35) |  |
|  |  | **p** | >0.999 | 0.059 | 0.285 | 0.135 | 0.094 | 0.370 |  |
| **Total proteins, n (%)** | | **BPD-DS** | 9 (42.9)  (n=21) | 10 (37.0)  (n=27) | 6 (37.5)  (n=16) | 5 (33.3)  (n=15) | 5 (41.7)  (n=12) | 3 (27.3)  (n=11) |  |
|  |  | **SADI-S** | 14 (26.4)  (n=53) | 23 (31.1)  (n=74) | 17 (27)  (n=63) | 15 (26.3)  (n=57) | 8 (25.8)  (n=31) | 8 (22.2)  (n=36) |  |
|  |  | **p** | 0.178 | 0.634 | 0.538 | 0.747 | 0.460 | 0.703 |  |
| **Iron, µg/dL** | | **BPD-DS** | 3 (14.3)  (n=21) | 5 (18.5)  (n=27) | 4 (25.0)  (n=16) | 3 (23.1)  (n=13) | 1 (8.3)  (n=12) | 3 (23.1)  (n=13) |  |
|  |  | **SADI-S** | 13 (24.5)  (n=53) | 8 (10.7)  (n=75) | 11 (16.2)  (n=68) | 9 (15.3)  (n=59) | 7 (18.9)  (n=37) | 4 (10.5)  (n=38) |  |
|  |  | **p** | 0.532 | 0.321 | 0.471 | 0.444 | 0.660 | 0.352 |  |

Supplementary Table 5. Comparison of nutritional deficiencies of patients submitted to submitted to biliopancreatic diversion with duodenal switch (BPD/DS) and single anastomosis duodeno-ileal bypass with sleeve gastrectomy (SADI-S) during a follow-up of 60 or more months

Fisher’s exact test was used to compare the number of patients with nutritional deficiencies between the surgical groups.
